# Tripartite Symbiotic Digestion of Lignocellulose in the Digestive System of a Fungus-Growing Termite

**DOI:** 10.1128/spectrum.01234-22

**Published:** 2022-10-17

**Authors:** Farhan Ahmad, Guiying Yang, Yaning Zhu, Michael Poulsen, Wuhan Li, Ting Yu, Jianchu Mo

**Affiliations:** a Ministry of Agriculture Key Lab of Molecular Biology of Crop Pathogens and Insect Pests, Institute of Insect Sciences, College of Agriculture and Biotechnology, Zhejiang University, Zhejiang, People’s Republic of China; b Entomology Section, Central Cotton Research Institute, Multan, Punjab, Pakistan; c Entomology Section, Central Cotton Research Institute, Sakrand, Shaheed Benazirabad, Sindh, Pakistan; d Section for Ecology and Evolution, Department of Biology, University of Copenhagen, Copenhagen East, Denmark; Howard University

**Keywords:** *Odontotermes formosanus*, bioconversion, lignocellulose, bacteria, *Termitomyces*, symbiosis

## Abstract

Fungus-growing termites are efficient in degrading and digesting plant substrates, achieved through the engagement of symbiotic gut microbiota and lignocellulolytic *Termitomyces* fungi cultivated for protein-rich food. Insights into where specific plant biomass components are targeted during the decomposition process are sparse. In this study, we performed several analytical approaches on the fate of plant biomass components and did amplicon sequencing of the 16S rRNA gene to investigate the lignocellulose digestion in the symbiotic system of the fungus-growing termite Odontotermes formosanus (Shiraki) and to compare bacterial communities across the different stages in the degradation process. We observed a gradual reduction of lignocellulose components throughout the process. Our findings support that the digestive tract of young workers initiates the degradation of lignocellulose but leaves most of the lignin, hemicellulose, and cellulose, which enters the fresh fungus comb, where decomposition primarily occurs. We found a high diversity and quantity of monomeric sugars in older parts of the fungus comb, indicating that the decomposition of lignocellulose enriches the old comb with sugars that can be utilized by *Termitomyces* and termite workers. Amplicon sequencing of the 16S rRNA gene showed clear differences in community composition associated with the different stages of plant biomass decomposition which could work synergistically with *Termitomyces* to shape the digestion process.

**IMPORTANCE** Fungus-farming termites have a mutualist association with fungi of the genus *Termitomyces* and gut microbiota to support the nearly complete decomposition of lignocellulose to gain access to nutrients. This elaborate strategy of plant biomass digestion makes them ecologically successful dominant decomposers in (sub)tropical Old World ecosystems. We employed acid detergent fiber analysis, high-performance anion-exchange chromatography (HPAEC), high-performance liquid chromatography (HPLC), scanning electron microscopy (SEM), Fourier transform infrared spectroscopy (FTIR), X-ray diffraction (XRD), pyrolysis gas chromatography-mass spectrometry (Py-GC-MS), and amplicon sequencing of the 16S rRNA gene to examine which lignocellulose components were digested and which bacteria were abundant throughout the decomposition process. Our findings suggest that although the first gut passage initiates lignocellulose digestion, the most prominent decomposition occurs within the fungus comb. Moreover, distinct bacterial communities were associated with different stages of decomposition, potentially contributing to the breakdown of particular plant components.

## INTRODUCTION

Lignocellulosic biomass is the most abundant nonfood renewable resource on Earth, composed of primarily polysaccharides (cellulose and hemicellulose) and lignin (1–3). Many phytophagous insects can decompose lignocellulose by secreting carbohydrate-active enzymes (CAZymes) (4–8). Insects, including leaf-cutting ants and termites, cannot produce all of the necessary enzymes to digest plant polymers themselves (9). The lignocellulose digestion in these insects requires complementary and synergistic cooperation between hosts and symbiotic microorganisms, particularly bacteria, flagellates, and fungi (3, 7, 10). Understanding these natural processes of lignocellulose digestion is promising to understand sustainable biological decomposition that has been optimized over millions of years of symbioses.

Fungus-growing termites, subfamily Macrotermitinae (Blattodea: Termitidae), are among the most successful phytophagous insects (11, 12). They can consume 20 to 90% of dead plant materials in tropical and subtropical areas of the world (13–15). More than 300 species of fungus-growing termites have been described, all of which engage in symbiotic association with multiple microbial symbionts, i.e., the gut microbiota, the fungal genus *Termitomyces* (Basidiomycota: Agaricales: Lyophyllaceae), and the bacterial community within external fungal combs (3, 16, 17). Among the herbivorous insects, these termites show comparatively higher rates of plant biomass decomposition, which appears to primarily stem from the mutualism with *Termitomyces* fungi (12, 18). These fungi can completely degrade and digest lignocellulosic materials, with consequent ecological impacts on ecosystem processes, particularly carbon cycling (19).

**FIG 1:**
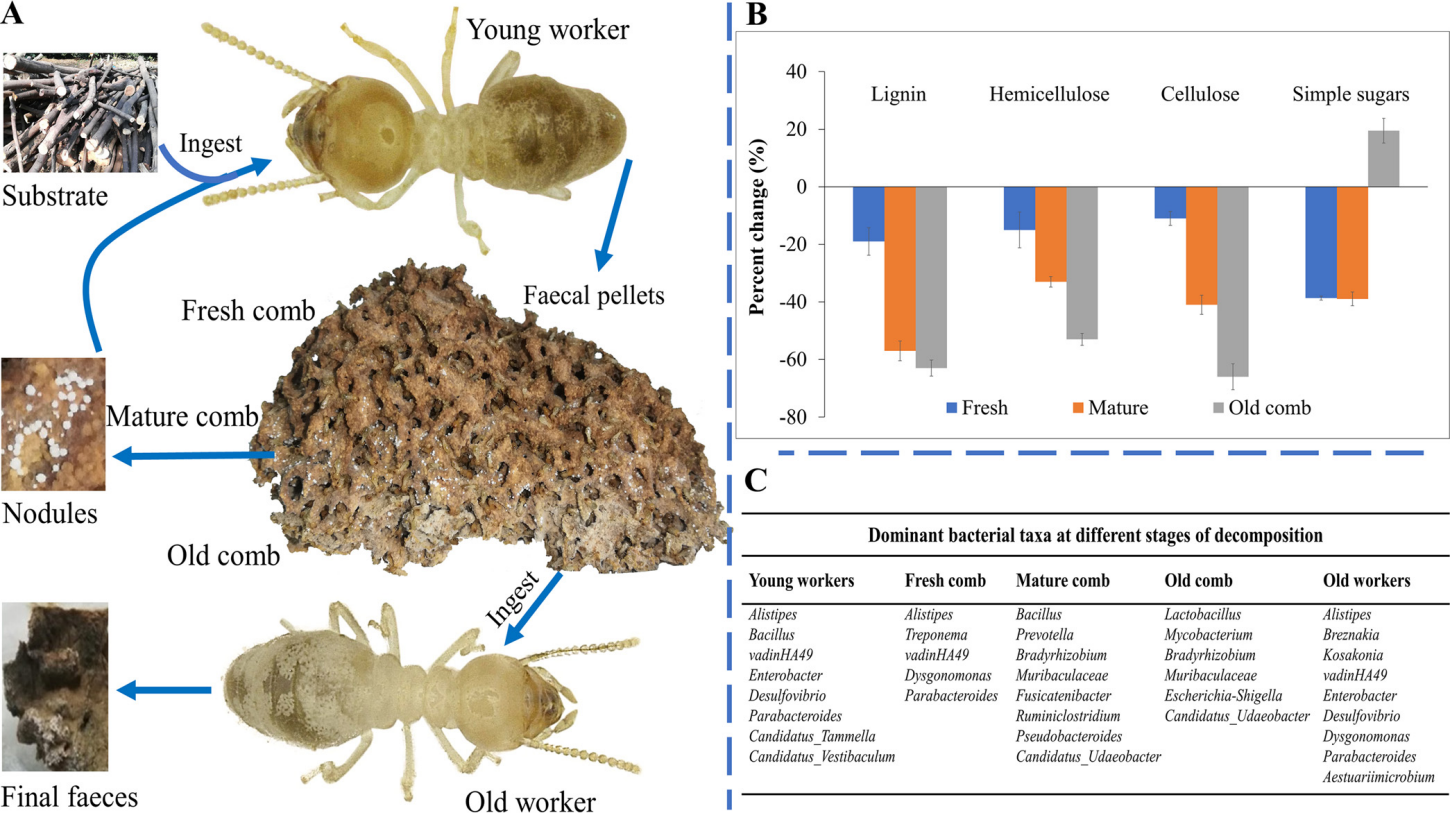
Summary figure providing a schematic representation of the lignocellulose digestion process in *Odontotermes formosanus* (A), a bar graph representing the percent change in plant components at different sites of decomposition relative to original mulberry wood (data from Fig. 2 and Table S1 in the supplemental material) (B), and the dominant bacterial taxa across the different stages of decomposition (C).

The symbiotic system (termite-bacterium-fungus) of these termites is sophisticated and allows the conversion of recalcitrant plant polysaccharides into simple monomers (20). The mechanism of lignocellulose decomposition involves a dual gut passage (Fig. 1A). Plant materials are foraged by the old termite workers and brought to the nest (7, 21). Young termite workers ingest this foraged material, along with fungal nodules (fungal structures in the mature parts of the fungus comb that hold asexual spores) (22, 23). The substrate passes through the gut (the so-called first gut passage) and is excreted as lignin-rich primary feces on the surface of the fungal comb. Minimum decomposition appears to occur during this first gut passage. *Termitomyces* spp. grow very fast on the primary feces and establish fresh fungus comb, which is then decomposed by the *Termitomyces* as the comb matures (24). In Odontotermes formosanus, after 4 weeks of additional degradation, the mature comb is converted into old comb, during which complex polysaccharides are broken down into simple sugars (12). This old comb is consumed by the old termite workers, and almost all carbohydrates are consumed in the so-called second gut passage (13).

Although some studies have investigated the degradation of plant biomass in fungus-growing termites (16, 21, 25–27), it is still unclear how abundant lignocellulose decomposition is across different stages of digestion. Contradictory results have been reported in the literature. For example, Leuthold et al. (28) and Aanen (29) reported that there is no digestion during the first gut passage because the young workers mainly use fungal nodules as a protein-rich food source. Contrary to this, an earlier study on digestion in Macrotermes michaelseni and Macrotermes subhyalinus by Veivers et al. (30) indicated cellulose digestion inside the digestive tract. Li et al. (27) also reported that strong lignin bonds are partly cleaved inside the gut of young workers. However, previous and some more recent studies indicate that the modification of lignin mainly takes place within the fungus comb (21, 24, 31).

A great diversity of bacteria has also been described in the guts (32–34) and fungus combs (35, 36). Although gut bacteria are consistently deposited to the fresh comb (35), there is a marked difference between the gut and comb microbiota (37). As the young workers ingest forage material and the old workers consume the mature comb, the changes in diet influence the bacterial composition of the gut (38). Early and recent studies on Macrotermes gilvus (39) and *O. formosanus* (37) reported that the gut community composition is affected by the differences in worker age. Furthermore, recently it was also reported that the bacterial taxa vary in combs of different age (40). It is well documented that the bacterial communities in the symbiotic system of fungus-growing termites are involved in atmospheric nitrogen fixation (36, 41, 42), reductive acetogenesis (12, 43), production of antimicrobial metabolites (44, 45), and contribution of fungus- and lignocellulose-degrading enzymes (7). For example, the members of *Bacteroidota* and *Firmicutes* in fungus-growing termite guts are dominant producers of CAZymes to degrade the fungus cell wall (33). *Proteobacteria* appear to be involved in nitrogen fixation (46, 47) and aromatic compound degradation (37). *Actinobacteriota* produce antimicrobial metabolites that may provide defense against invading fungal species (44), and they are also capable of breaking down polysaccharides (45, 48). *Synergistota* are considered amino acid-degrading bacteria (41). *Spirochaetota* are generally infrequent in fungus-growing termites (49, 50), but they may be involved in reductive acetogenesis and nitrogen fixation (51). Despite this, we have a generally poor understanding of where different plant components are targeted during the stages in the decomposition process and the presence and hence potential importance of specific bacterial community members in the processes.

Here, we used fiber detergent analysis (FDA), high-performance anion-exchange chromatography (HPAEC), high-performance liquid chromatography (HPLC), scanning electron microscopy (SEM), Fourier transform infrared spectroscopy (FTIR), X-ray diffraction (XRD), and pyrolysis gas chromatography-mass spectrometry (Py-GC-MS) to investigate the lignocellulose decomposition in the symbiotic system of *O. formosanus*. We complement this with amplicon sequencing of the 16S rRNA gene to characterize bacterial communities throughout the degradation process. Our findings documented the consistent reduction of lignocellulosic components at different stages of decomposition (Fig. 1B) and the distinct bacteria across different sites of degradation (Fig. 1C). The results of the present work will help us to better understand the digestive process in fungus-growing termites.

## RESULTS

### Comparative lignocellulose content analyses throughout the decomposition process.

**FIG 2:**
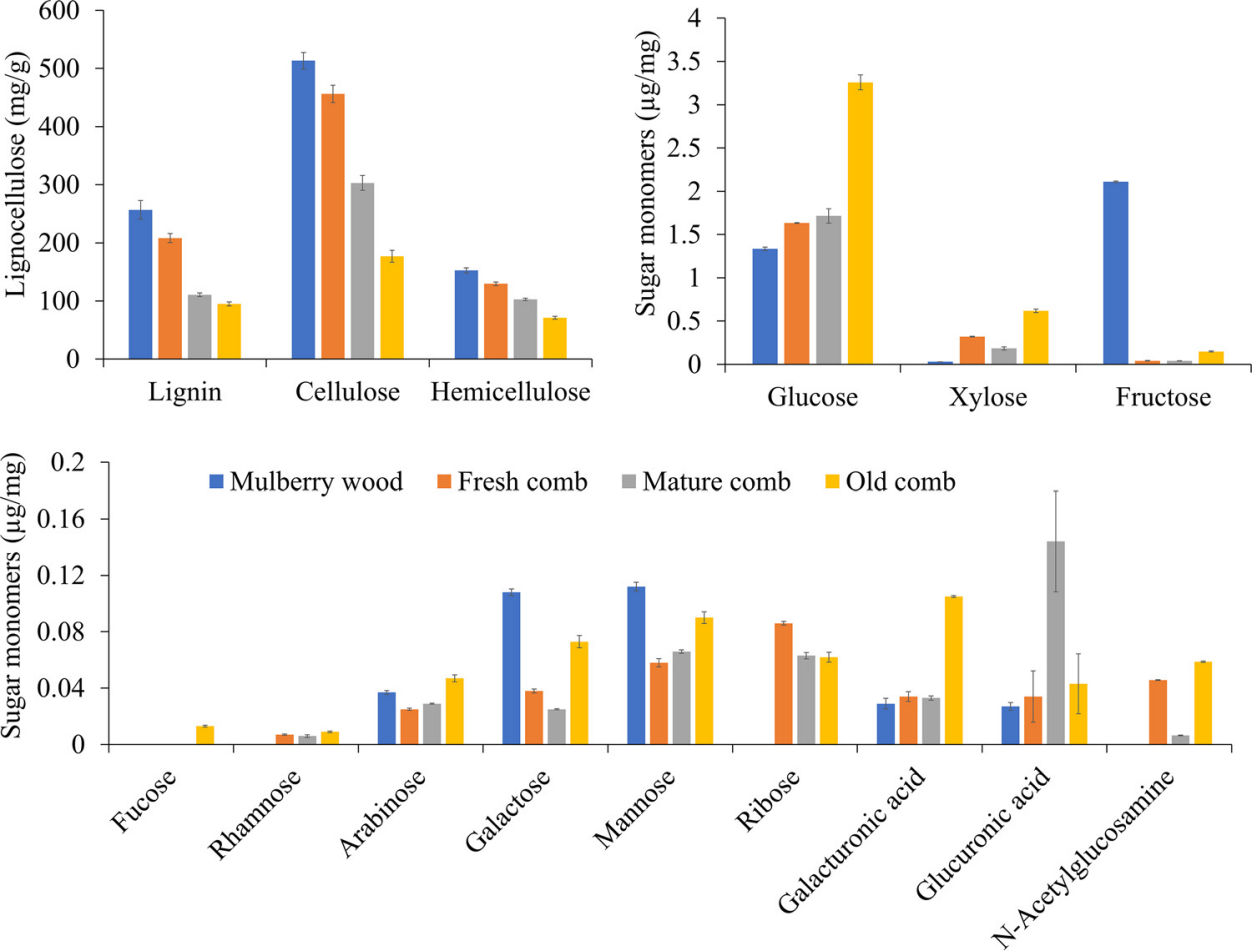
Lignocellulose composition across different stages in the decomposition process. Error bars indicate standard deviation (*n* = 3 colonies).

We first compared the digested lignocellulose samples (fresh, mature, and old comb) from three colonies with original mulberry wood by lignocellulose compositional analysis using the fiber detergent method (52). The original contents of lignin, cellulose, and hemicellulose in mulberry wood were 256.6, 513.3, and 152.5 mg/g, respectively, consistent with previous work (53). The analysis of comb material revealed that lignocellulosic components were significantly degraded in all three colonies (Fig. 2). Specifically, lignin, cellulose, and hemicellulose were on average reduced by 18.9%, 11.1%, and 15.0% in the fresh comb, 56.9%, 41.0%, and 32.5% in the mature comb, and 63.0%, 65.5%, and 53.4% in old comb, respectively (see Table S1 in the supplemental material).

We then employed high-performance anion-exchange liquid chromatography (HPAEC) (54) and high-performance liquid chromatography (HPLC) to determine 14 important monomeric sugars in mulberry wood and digested combs (fresh, mature, and old comb). Galactose, mannose, and fructose were significantly reduced by 64.8%, 48.2%, and 98.1% (fresh comb), 76.9%, 41.1%, and 98.1% (mature comb), and 32.4%, 19.6%, and 92.9% (old comb), respectively. Arabinose also decreased in fresh (32.4%) and mature (21.6%) comb but increased in the old comb (27.0%). In contrast, xylose, glucose, and galacturonic and glucuronic acid significantly increased by 966.7%, 22.3%, 17.2%, and 25.9% in fresh comb, 513.3%, 28.5%, 13.8%, and 433.3% in mature comb, and 1,956.7%, 144.0%, 262.1%, and 59.3% in the old comb, respectively. Rhamnose, ribose, and *N*-acetylglucosamine sugars were not found in the control sample, but they were detected in combs. Fucose was detected only in the old comb. We did not detect guluronic acid and mannuronic acid. This means that the diversity of simple sugars was higher in the three comb categories than in wood as the control. While the total amount of monomeric sugars reduced in fresh and mature comb, they generally increased in the old comb (Table S1). This implies that the decomposition process makes monomers available after the nearly complete degradation of complex carbohydrates within the comb.

### Characterization of lignocellulose surface differences across decomposition stages.

**FIG 3:**
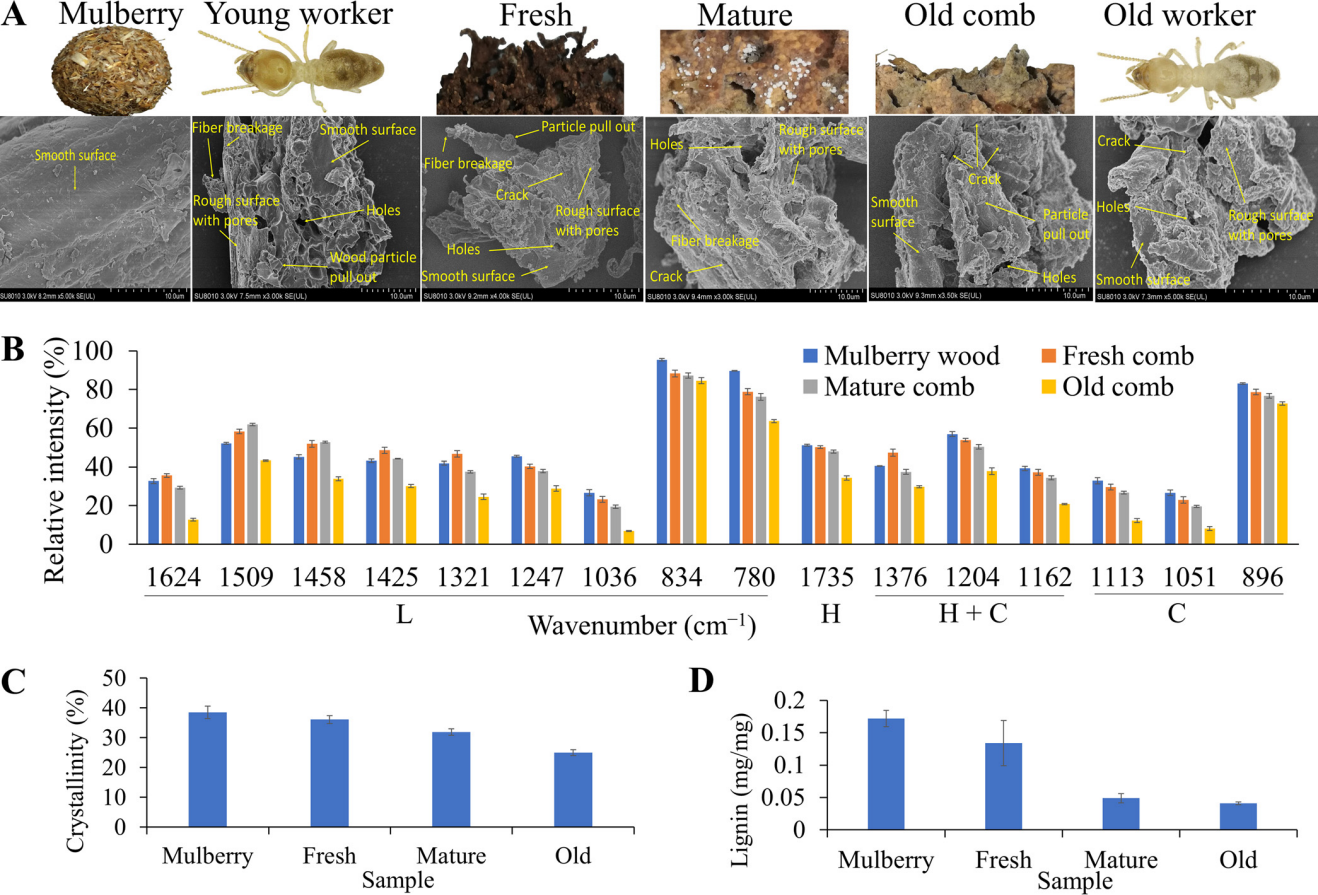
(A) SEM images of the original mulberry wood, a gut of a young worker, fresh comb, mature comb, old comb, and the gut of an old worker; (B) determination of band intensities by using FTIR (bands with corresponding bond vibrations and functional groups are listed in Table S2 in the supplemental material); (C) quantification of crystallinity index by using XRD; (D) confirmation of lignin degradation by using pyrolysis-GC-MS. Error bars indicate standard deviation. L, lignin; H, hemicellulose; C, cellulose. *n* = 3 colonies.

The surface morphologies of three comb categories (fresh, mature, and old comb), young and old worker guts, and original wood were compared using scanning electron microscopy (SEM). Representative SEM images of treated and untreated samples are shown in Fig. 3A. The surface of the original wood was morphologically tight, orderly, smooth, and intact. However, the wood particles obtained from comb samples and termite guts showed ultrastructure disturbances in support of the structural changes associated with their breakdown.

### Characterization of chemical structures by FTIR analysis.

To provide further insights into structure-level changes in lignin, hemicellulose, and cellulose throughout the decomposition process, we performed Fourier transform infrared spectroscopy (FTIR) (55). The FTIR spectra of mulberry wood, fresh comb, mature comb, and old comb are given in Fig. 3B. Although this region comprises the 4,000 to 500 cm^−1^ wavenumber range, the region of 1,800 to 750 cm^−1^ contains the most spectral information (Fig. S1A). The recorded bands correspond to the lignocellulosic components given in Table S2. Within the fingerprint region, 16 bands were monitored. Compared to mulberry wood, the FTIR spectra of fresh, mature, and old fungus comb revealed a gradual diminution in peaks 1,735 cm^−1^ (hemicellulose), 1,376 cm^−1^, 1,204 cm^−1^, and 1,162 cm^−1^ (hemicellulose and cellulose), 1,051 cm^−1^ (cellulose), 1,113 cm^−1^ (crystalline cellulose), and 896 cm^−1^ (amorphous cellulose) at all decomposition stages. This indicates a stepwise degradation of hemicellulose, crystalline cellulose, and amorphous cellulose throughout the process. Changes in the intensities of lignin-associated peaks were also observed. For example, 1,624 cm^−1^, 1,425 cm^−1^, and 1,321 cm^−1^ were higher in fresh comb and decreased in mature and old comb. The peaks 1,509 cm^−1^ and 1,458 cm^−1^ were higher in fresh and mature comb and reduced only in old comb. Furthermore, 1,247 cm^−1^, 834 cm^−1^, and 780 cm^−1^ were consistently reduced in all three comb categories compared to original mulberry. These are clear indications of structural modifications of lignin at different stages of decomposition.

### Measurements of cellulosic crystallinity by XRD analysis.

Crystallinity index (CrI) is an important factor affecting the hydrolysis of cellulose. The crystallinity index of plant-based materials can be determined by using X-ray diffraction (XRD) (56). XRD spectra were obtained from the original mulberry and comb samples (Fig. S1B). Our results showed that the crystallinity index was reduced from 38.5% in mulberry wood to 36.1%, 31.9%, and 25.0% in fresh comb, mature comb, and old comb, respectively (Fig. 3C). The consistent reduction of crystallinity index throughout the decomposition process corresponded to the decrease of band 1,113 cm^−1^ in the FTIR spectra. Furthermore, the Scherrer equation (crystallite size = kλ/b cos θ) (57) also revealed a gradual reduction of crystallite size in fresh comb (2.988 nm), mature comb (2.176 nm), and old comb (1.080 nm) compared to original mulberry (3.294 nm). This confirms that the symbiotic system of fungus-growing termites efficiently decrystallizes crystalline cellulose.

### Confirmation of lignin degradation by Py-GC-MS.

Pyrolysis gas chromatography-mass spectrometry (Py-GC-MS) (1) was employed to confirm the degradation and depolymerization of lignin units in the symbiotic system of *O. formosanus*. The total number of peaks was 29 (fresh), 24 (mature), and 23 (old) compared to 36 in mulberry (Fig. S2). These results indicate that there is a continuous separation of polysaccharides from lignin during decomposition. The relative abundances and identities of the released phenolic compounds are listed in Table 1 and Fig. S3. Lignin content reduced from 0.172 in the mulberry sample to 0.134 (22% reduction), 0.049 (72%), and 0.041 (76%) in fresh, mature, and old comb, respectively (Fig. 3D). This is consistent with the detergent fiber analysis and FTIR spectra (Fig. 2 and 3B).

**TABLE 1:** Relative molar abundances of compound peaks identified in Py-GC-MS of the original mulberry wood and fungus combs at different ages

No.	Compound	Lignin type^*a*^	Relative molar abundance (%) in wood or comb			
			Original mulberry	Fresh comb	Mature comb	Old comb
1	Benzaldehyde		0.43 ± 0.16	0.64 ± 0.01	1.33 ± 0.21	1.38 ± 0.32
2	Phenol	H/C	1.54 ± 0.18	3.09 ± 0.71	5.86 ± 1.01	3.32 ± 0.22
3	2-Cresol	H	1.13 ± 0.07	1.47 ± 0.15	2.34 ± 0.41	2.72 ± 0.41
4	*p*-Cresol	H	1.06 ± 0.05	3.20 ± 0.0	3.06 ± 0.10	4.57 ± 0.43
5	Guaiacol	G	5.41 ± 0.36	6.69 ± 0.19	14.07 ± 0.13	15.81 ± 0.35
6	2,4-Dimethylphenol	H	1.03 ± 0.29	1.40 ± 0.01	2.42 ± 0.55	2.79 ± 0.59
7	4-Ethylphenol	H	0.49 ± 0.04	1.85 ± 0.01	0.00 ± 0.0	0.00 ± 0.0
8	2-Ethylphenol	H	0.49 ± 0.09	0.00 ± 0.0	0.00 ± 0.0	0.00 ± 0.0
9	Creosol	G	2.20 ± 0.17	4.75 ± 1.29	2.05 ± 0.25	3.69 ± 0.55
10	4-Vinylphenol		7.91 ± 0.64	7.77 ± 2.46	0.00 ± 0.0	0.00 ± 0.0
11	Catechol		3.43 ± 0.37	5.02 ± 0.48	6.06 ± 0.10	4.01 ± 0.24
12	3-Methoxycatechol		2.12 ± 0.0	1.68 ± 0.16	0.00 ± 0.0	0.00 ± 0.0
13	4-Ethylguaiacol	G	2.83 ± 0.16	2.26 ± 0.47	1.81 ± 0.53	0.00 ± 0.0
14	4-Vinylguaiacol	G	9.09 ± 0.30	8.69 ± 0.73	4.72 ± 1.33	6.56 ± 0.83
15	Syringol	S	6.43 ± 0.05	6.12 ± 0.35	6.93 ± 0.08	11.89 ± 1.27
16	Eugenol	G	3.07 ± 0.11	1.77 ± 0.06	1.44 ± 0.02	0.84 ± 0.20
17	3,4-Dimethoxyphenol	S	0.79 ± 0.05	2.29 ± 0.86	0.00 ± 0.0	0.00 ± 0.0
18	Vanillin	G	1.47 ± 0.0	2.30 ± 0.05	2.24 ± 0.37	5.10 ± 0.80
19	1,2,3-Trimethoxybenzene	S	0.00 ± 0.0	4.09 ± 0.29	4.29 ± 0.16	5.40 ± 0.16
20	Isoeugenol	G	2.79 ± 0.04	0.00 ± 0.0	0.00 ± 0.0	0.00 ± 0.0
21	Vanillic acid	G	2.71 ± 0.71	3.99 ± 0.34	2.14 ± 0.04	1.92 ± 0.27
22	*trans*-Isoeugenol	G	7.99 ± 0.35	4.21 ± 0.17	2.12 ± 0.09	1.28 ± 0.33
23	Apocynin	G	2.51 ± 0.31	3.51 ± 0.14	4.42 ± 0.23	2.04 ± 0.27
24	Vanillic acid methyl ester	G	0.00 ± 0.0	0.00 ± 0.0	0.00 ± 0.0	4.60 ± 0.39
25	4-Ethyl-2,6-dimethoxyphenol	S	2.22 ± 0.10	2.78 ± 0.38	6.29 ± 0.52	3.12 ± 0.69
26	Homovanillic acid	G	2.29 ± 0.16	4.42 ± 0.80	10.94 ± 0.17	4.81 ± 0.04
27	Methyleugenol	G	2.79 ± 0.11	0.00 ± 0.0	0.00 ± 0.0	0.00 ± 0.0
28	4-Allylsyringol	S	4.60 ± 0.24	3.72 ± 0.51	2.54 ± 0.18	0.00 ± 0.0
29	Acetoeugenol	G	0.54 ± 0.20	0.00 ± 0.0	0.00 ± 0.0	0.00 ± 0.0
30	Syringyl vinyl ketone	S	0.56 ± 0.04	0.00 ± 0.0	0.00 ± 0.0	0.00 ± 0.0
31	Syringe aldehyde	S	2.43 ± 0.21	3.88 ± 0.29	3.98 ± 0.92	2.87 ± 0.53
32	1-(3,5-Dimethoxy-4-hydroxyphenyl) propyne	S	1.85 ± 0.19	1.33 ± 0.05	0.00 ± 0.0	0.00 ± 0.0
33	4-Acetylsyringol	S	2.03 ± 0.13	3.93 ± 0.66	4.12 ± 0.13	5.04 ± 1.02
34	*cis*-Coniferyl alcohol	G	7.26 ± 0.52	0.00 ± 0.0	0.00 ± 0.0	0.00 ± 0.0
35	Syringylacetone	S	1.56 ± 0.01	2.23 ± 0.08	3.23 ± 0.29	4.34 ± 0.12
36	*trans*-Sinapaldehyde	S	0.46 ± 0.03	0.00 ± 0.0	0.00 ± 0.0	0.00 ± 0.0
37	Propiosyringone	S	0.42 ± 0.03	0.93 ± 0.04	1.59 ± 0.12	1.89 ± 0.12
38	*trans*-Sinapyl alcohol	S	3.47 ± 0.54	0.00 ± 0.0	0.00 ± 0.0	0.00 ± 0.0

	Lignin monomer (mg)	H	0.007	0.011	0.004	0.004
		G	0.092	0.057	0.022	0.019
		S	0.046	0.042	0.016	0.014
	Total lignin content (mg) from 1-mg sample^*b*^		0.172	0.134	0.049	0.041
	% reduction			22%	72%	76%

a H, hydroxyphenyl unit; C, carbohydrates; S, syringyl unit; G, guaiacyl unit.

b Total lignin was calculated based on the percentage of internal standard in all lignin degradation products.

The pyrograms of the fungus comb samples and original wood revealed compounds derived from *p*-hydroxyphenyl, guaiacyl, and syringyl. The compounds with one or two methoxy substituents *ortho* to the phenolic hydroxyl group were considered G or S lignin, respectively, while the compounds with no methoxy substituents were deemed H lignin (58). The H-lignin units were 0.011, 0.004, and 0.004 in fresh, mature, and old comb, respectively, compared to 0.007 in mulberry wood. The G-lignin monomers were 0.057, 0.022, and 0.019 compared to 0.092 in the control sample. The S-lignin monomers were 0.042, 0.016, and 0.014 compared to 0.046 in mulberry (Table 1).

### Characterization of the bacterial community in the symbiotic system.

**FIG 4:**
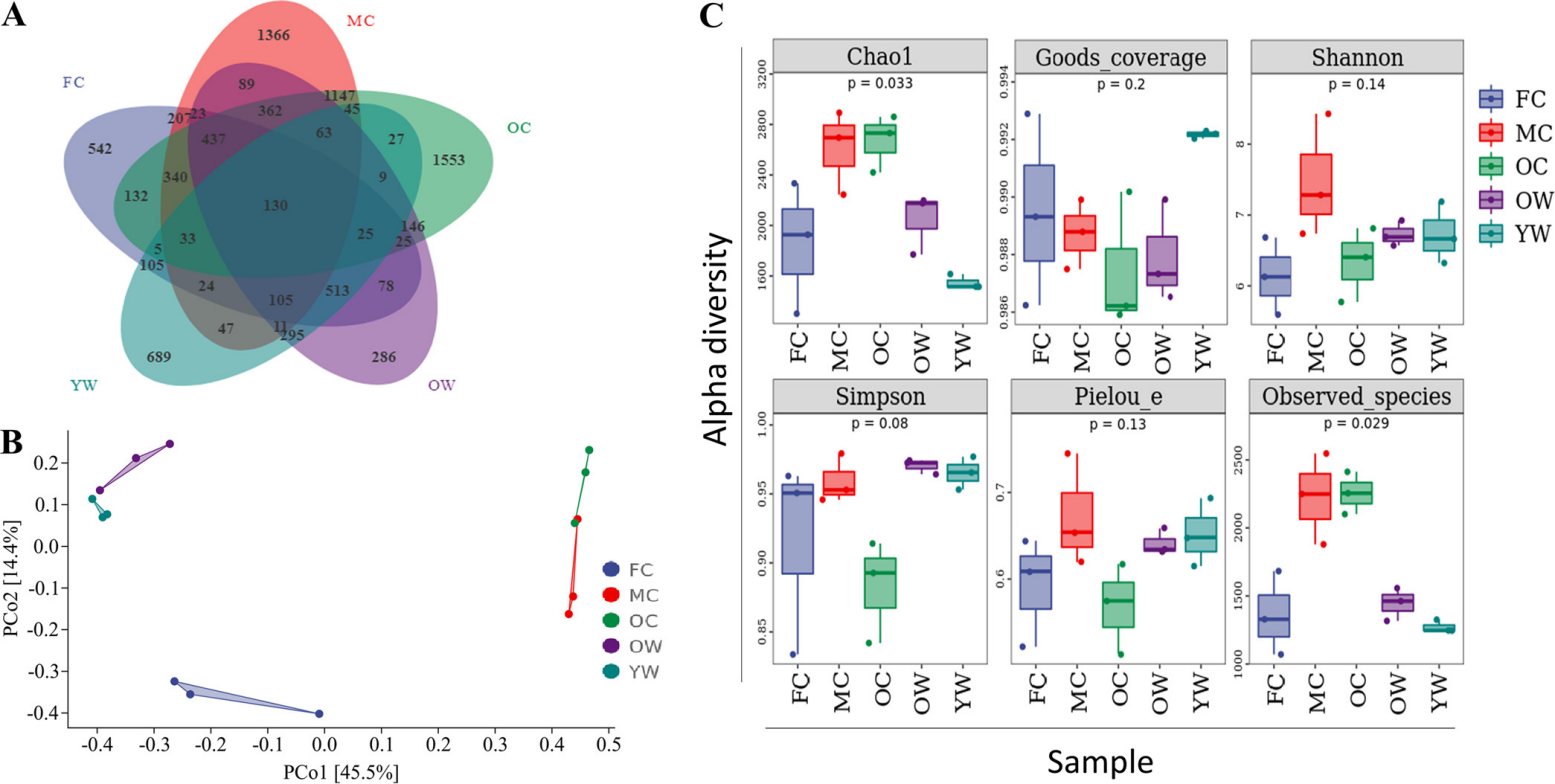
Bacterial community profiles across different stages in the decomposition process. (A) Venn diagram of OTU showing the overlap of genera among different groups. (B) Bacterial community similarity analysis of termite guts and three comb categories, visualized using principal-coordinate analysis (PCoA). (C) Box-and-whisker plots demonstrated significance among five stages of decomposition in different indicators, including the number of sequences, richness, coverage, evenness, and diversity. FC, fresh comb; MC, mature comb; OC, old comb; OW, old workers; YW, young workers. Results of Kruskal-Wallis test and Dunn’s *post hoc* tests for multiple comparison are given within plots. *n* = 3 colonies.

Amplicon sequencing of the 16S rRNA gene (36, 38) was applied to investigate and compare bacterial community compositions across the stages in the decomposition process. We obtained a total of 103,740 to 148,217 high-quality reads per sample (Table S4). A Venn diagram comparison illustrated that fresh comb, mature comb, old comb, young worker, and old worker occupied 542, 1,366, 1,553, 689, and 286 unique operational taxonomic units (OTUs), respectively, while only 130 OTUs were shared across all samples (Fig. 4A). Principal-coordinate analysis (PCoA) separated the bacterial communities into three main groups (Fig. 4B), with fresh comb samples being separated from a cluster of mature and old comb and the third cluster harboring young and old workers. Bacterial communities in workers were thus more similar to each other and distinct from those in comb. Microbial richness was significantly different between groups, when evaluated using both the Chao1 (*P* < 0.05) and observed species (*P < *0.05) indices. Mature and old comb had the highest species richness (Table S4). Microbial diversity (Shannon and Simpson) and evenness (Pielou’s evenness) were not significantly different between groups (Fig. 4C).

**FIG 5:**
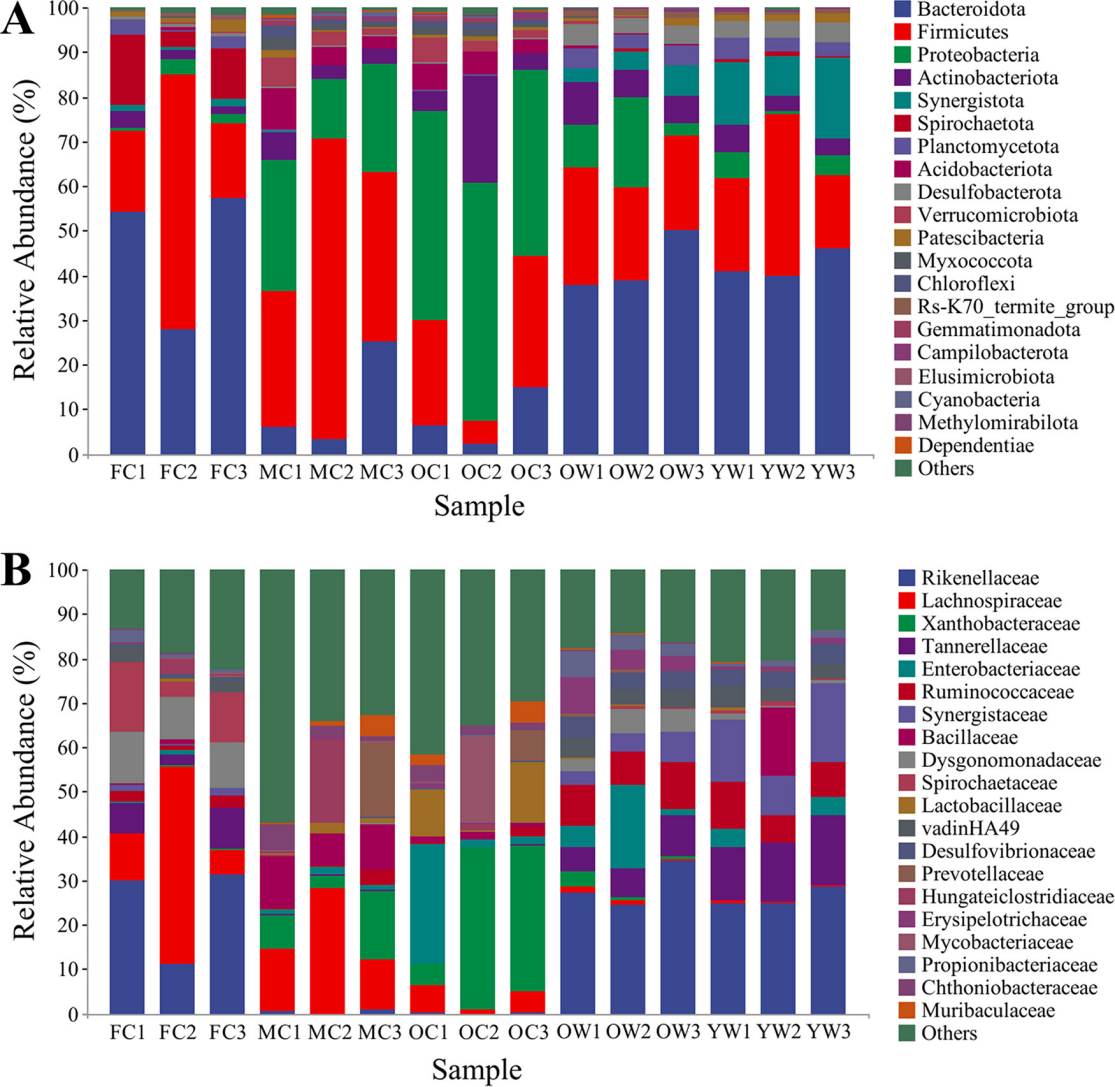
Comparison of bacterial community structure throughout the decomposition process. (A) Phylum-level comparison of bacterial OTUs. (B) Relative abundance of bacteria at the family level (*n* = 3 colonies).

Twenty bacterial phyla were identified, and the most prominent ones were *Bacteroidota* (2.24% to 57.39% relative abundance), *Firmicutes* (5.28% to 67.17%), *Proteobacteria* (0.38% to 53.23%), *Actinobacteriota* (1.61% to 23.87%), *Synergistota* (0% to 17.9%), and *Spirochaetota* (0% to 15.69%) (Fig. 5; Tables S5 and S6). *Rikenellaceae*, *Tannerellaceae* (*Bacteroidota*), *Ruminococcaceae* (*Firmicutes*), and vadinHA49 (*Planctomycetota*) were abundantly represented in fresh comb and workers, whereas *Xanthobacteraceae* (*Pseudomonadota*), *Prevotellaceae*, *Muribaculaceae* (*Bacteroidota*), and *Chthoniobacteraceae* (*Verrucomicrobiota*) were more abundant in mature and old comb (Table S6). *Ruminococcaceae* (*Firmicutes*) were relatively stable across samples, while *Lachnospiraceae* (*Firmicutes*) were most common in fresh and mature comb. *Enterobacteriaceae* (*Proteobacteria*) were abundant in old comb and old workers, whereas *Synergistaceae* (*Synergistota*) and *Desulfovibrionaceae* (*Proteobacteria*) were relatively more abundant in workers than in combs. Likewise, *Dysgonomonadaceae* (*Bacteroidota*) and *Spirochaetaceae* (*Spirochaetota*) were relatively high in abundance in fresh comb, whereas *Bacillaceae* and *Hungateiclostridiaceae* (*Firmicutes*) were more abundant in mature comb. *Lactobacillaceae* (*Firmicutes*) and *Mycobacteriaceae* (*Actinobacteriota*) were most abundantly represented in old comb (Fig. 5B and Table S6).

**FIG 6:**
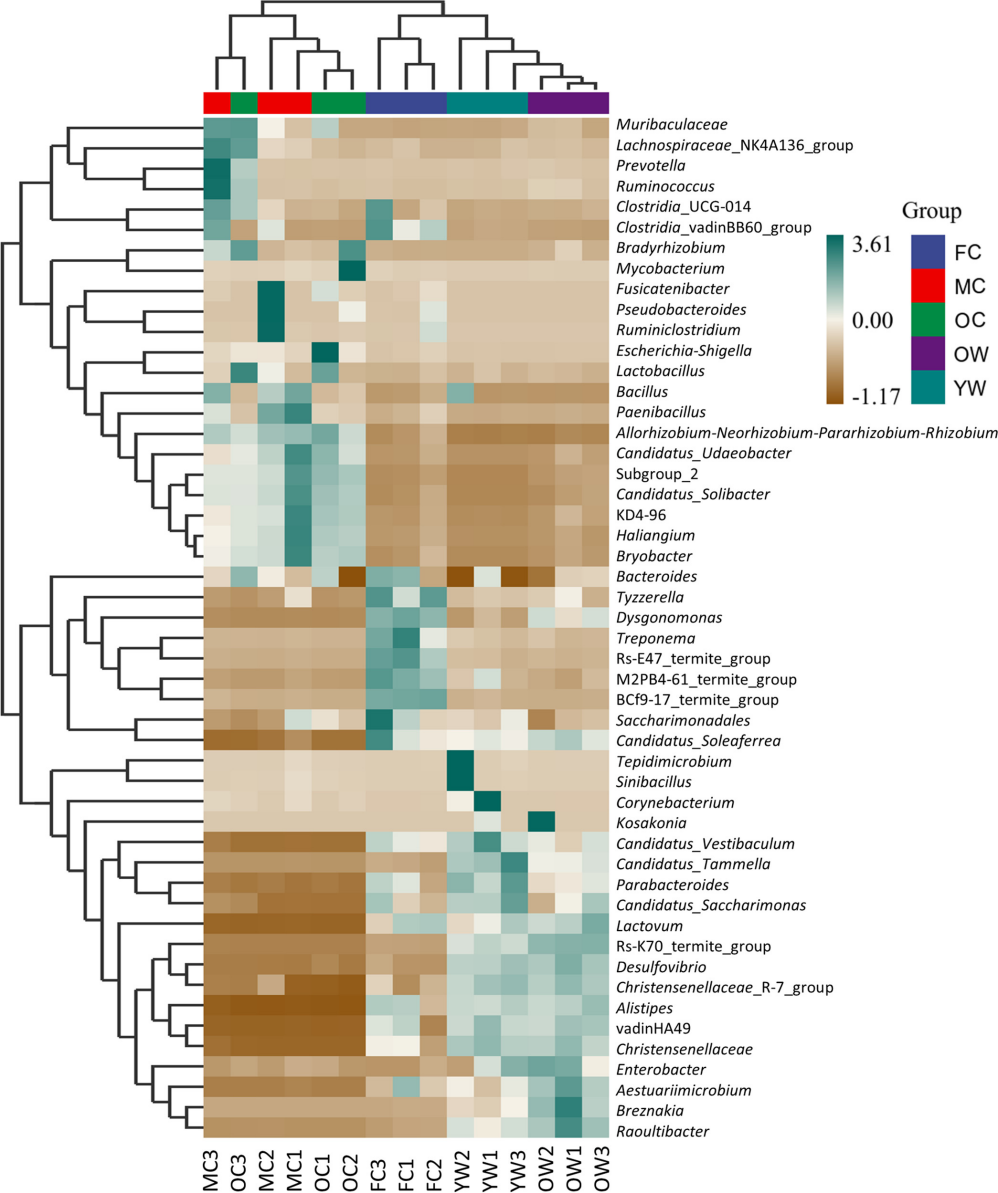
A heatmap of bacterial taxa (accounting for >1% of relative abundance in at least one sample) enriched (teal green) or contracted (brown) in relative abundance across different stages in the decomposition process, with scale bar indicating percentages ranging from −1.17 to 3.61% relative abundance differences. The horizontal labels give sample identifiers (IDs) (mentioned in Fig. 4), and the vertical labels represent the bacterial taxa at genus level (see Table S7 in the supplemental material). Cluster analysis at the top shows similarities and differences of community compositions of different samples, indicating that the relative abundances of workers’ core bacterial taxa are slightly similar to fresh comb communities while entirely different from the communities associated with mature and old combs (*n* = 3 colonies).

**FIG 7:**
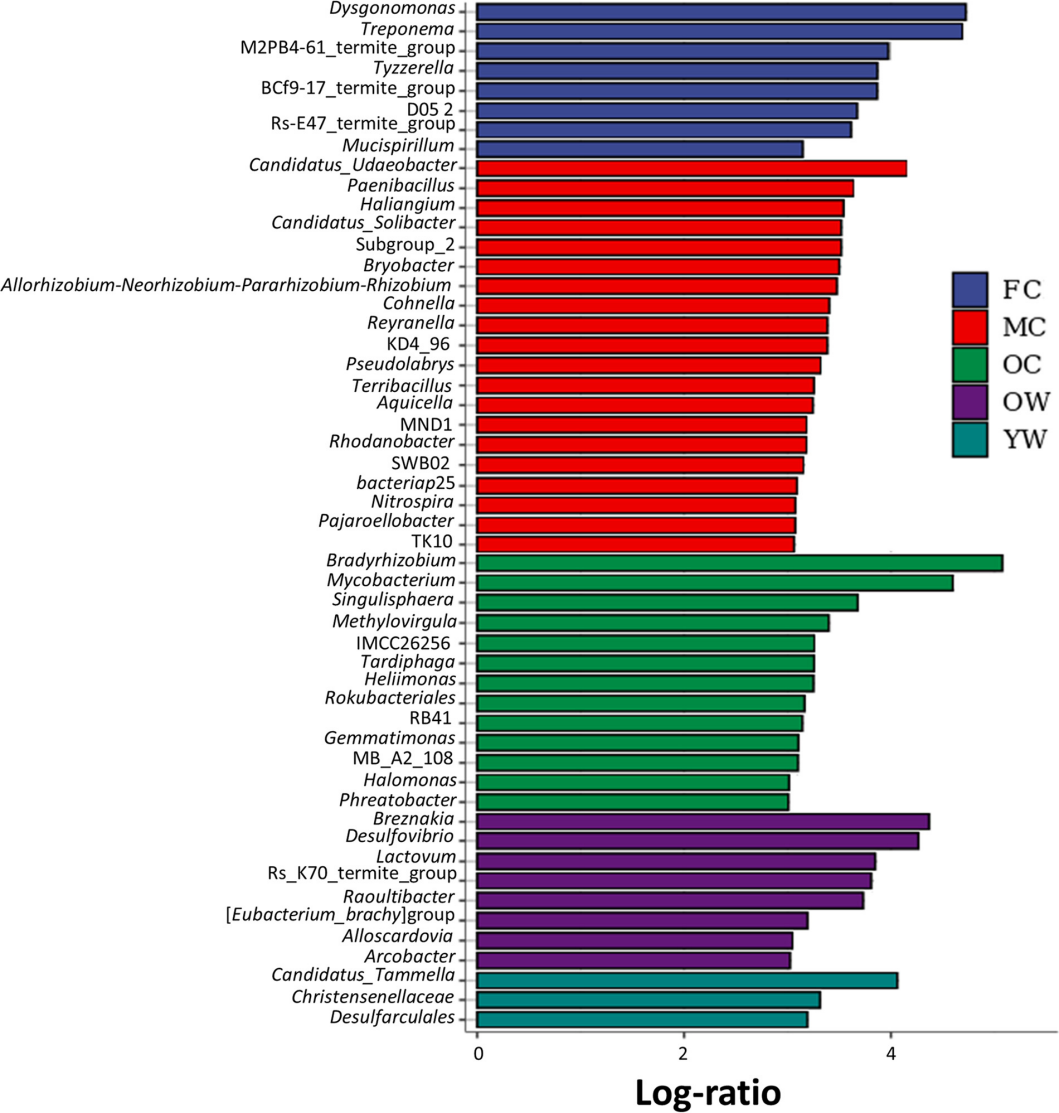
Linear discriminant analysis (LDA) effect size (LEfSe) of termite guts and fungus combs shows different abundances throughout the decomposition process. Horizontal bars represent the effect size for each taxon. LDA score is greater than 3.0. The taxon of bacteria with significant change (*P* < 0.05) in relative abundance is written alongside the horizontal lines. *n* = 3 colonies.

Linear discriminant analysis effect size (LEfSe) analysis demonstrated that bacterial communities in worker guts were different from those of combs. For instance, members of “*Candidatus* Tammella” (*Synergistota*) were comparatively high in young workers, whereas *Breznakia* (*Bacillota*) and *Desulfovibrio* (*Pseudomonadota*) were predominantly found in old workers. *Dysgonomonas* (*Firmicutes*) and Treponema (*Spirochaetota*) were significantly more abundant in fresh comb (Fig. 6 and 7; Table S7). Moreover, “*Candidatus* Udaeobacter” (*Verrucomicrobiota*) was most abundant in mature comb, while *Bradyrhizobium* (*Pseudomonadota*) and Mycobacterium (*Actinobacteriota*) were predominantly found in the old comb (Fig. 6 and 7; Table S7).

## DISCUSSION

The degradation of lignocellulose is central to the success of fungus-growing termites. Although many reports have documented the roles of especially *Termitomyces* in the decomposition process (21, 24, 27–31), our understanding of the process and mechanisms of decomposition in the symbiotic system remains limited. Our findings provide new insights into the stages of the process of plant biomass digestion in *Odontotermes formosanus*.

### Which lignocellulose components are decomposed in the process?

Our analytical approaches showed that the symbiotic system of *O*. *formosanus* has the ability to degrade lignocellulosic components efficiently and support the idea that gut passage through young workers, to some extent, initiates this degradation process, consistent with previous work (27, 30). However, the ample amount of lignin, hemicellulose, and cellulose in the fresh comb is a clear indication that the fungus comb is the main location for decomposition of plant biomass, consistent with previous results (21, 24). However, it is noteworthy that the intricate details of the process, and the magnitude of change in plant structural components in young worker guts, may vary by termite species (59), so that more extensive comparative analyses across the phylogeny of the subfamily are warranted to shed light on how conserved this pretreatment process is.

The increases in aromatic compounds, such as phenol (peak 2), 2-methoxyphenol (peak 5), and 2,6-dimethoxyphenol (peak 15), in the comb samples indicated a stepwise depolymerization of lignin. Guaiacyl and syringyl lignin monomers were gradually reduced, whereas the *p*-hydroxyphenyl lignin units increased in fresh comb and later decreased in mature and old comb. The scanning electron microscopy images revealed physical disruption of the lignocellulose structure at both gut and comb stages, consistent with a role of guts and fungus comb, but most lignin remained intact in the fresh comb and only significantly decreased in mature and older parts of the fungus comb (21, 24). This suggests that even if the process of lignin cleavage is initiated in the gut, it is likely to only be due to the mechanical treatment that is associated with the maceration of plant substrate and less likely to represent enzymatic or oxidative processes.

We found an increased diversity of simple sugars in the comb compared to mulberry wood, but there was a reduced amount of monomeric sugars in fresh and mature comb that increased only in old comb. The decomposition process thus results in old comb food for the termites that is enriched with simple carbohydrates, particularly glucose, xylose, arabinose, ribose, galacturonic acid, and *N*-acetylglucosamine. This is consistent with previous work (21, 30, 60). Galacturonic acid is the main component of pectin from the plant substrate. The large amount of this sugar acid in old fungus comb suggests that *Termitomyces* decomposes pectin efficiently and enriches the old comb with galacturonic acid. da Costa et al. (21) also reported ample amounts of this sugar acid in the fungus comb, and Pramanik and Islam (61) also isolated it from Termitomyces eurhizus.

As the old comb is ingested by the old workers, the fungal biomass and undigested simple sugars enter the gut, where bacteria digest and ferment them to short-chain fatty acids (33), which can be utilized by the host. This implies that the fungal biomass and the bacteria in the gut provide nutrition to the termites during the second gut passage. To gain a more fundamental understanding of the role of fungal and sugar sources in digestion, further metabolomic work using novel approaches such as labeling of specific nutritional compounds could shed light on which lignocellulose components are utilized by the fungus, bacteria, and termites, respectively.

Although the lignocellulose degradation mechanism in this study is in the line with previous reports (21, 27), we did observe quantitative differences in lignocellulose contents at different stages of decomposition that differ from previous work (see comparisons in Table S3 in the supplemental material). This may be due to the use of different methods and termite species, but it would be interesting to explore if other factors underpin the discrepancies between our results and those reported by da Costa et al. (21), who explored decomposition of the plant substrate in nature while our samples were from captive colonies.

### Distinct bacterial communities across different stages of decomposition.

The amplicon sequencing analysis provided new insights into bacterial composition in the symbiotic system of *O. formosanus*. We found diverse and distinct bacterial communities throughout the degradation process of this species. As expected from previous work (35, 37, 38, 40), we observed major differences in community composition between workers and fungus comb, reflecting different environmental conditions and microbial processes. Although most sequence reads were unique in workers or comb, several taxa belonged to shared bacterial lineages. Particularly bacterial taxa in the fresh fungus comb were often also identified in the guts, from which they are at least in part derived (35). In contrast to this, the dominant bacterial communities in mature and old comb were entirely absent from worker guts. These bacteria may thus be from the surrounding soil environment or be too infrequent in the gut to amplify during sequencing. These observations indicate that the roles and impact of bacteria on decomposition and other functions are, as expected, likely to be vastly different between guts and combs (cf. references 37 and 50).

Most bacterial taxa (particularly *Alistipes*) were consistent in relative abundances between young and old workers, suggesting similar microenvironmental conditions and microbial processes, although gene expression may vary. However, we did find slight differences in the abundances of certain gut microbes between young and old workers, which may be due to the type of the ingested diet, which is the plant substrate and fungal nodules in young workers but predominantly fungus comb in old workers (38, 62). Hongoh et al. (39) reported that the differences in age of fungus-growing termite workers indeed affect microbial community compositions within guts. The higher relative abundances of *Breznakia* in old workers and “*Candidatus* Tammella” in young workers (Fig. 7) were the clearest difference observed, which may be associated with the changes in the plant components, particularly the high consumption of simple sugars and protein by old workers. It is noteworthy that members of *Breznakia* that were abundant in old workers (this study) are also abundantly represented in cockroaches and other insects (63). Breznakia blatticola and Breznakia pachnodae are considered fermenters of glucose to formate, ethanol, and acetate (63, 64). The increased abundance of the genus “*Candidatus* Tammella” has also been recorded in the gut of Macrotermitinae (37, 50) and dry wood termites (65), and it has been suggested that some members of this genus are involved in amino acid fermentation (65).

The marked differences in community composition between fresh and older (mature and old comb) parts of the comb are intriguing, likely shaped by continuous gut deposits versus input from the surrounding soil. Young workers transport a diversity of bacteria to fresh comb (35) of which some, but not all, persist in older sections of the combs. Here, communities are further impacted by the surrounding mound soil (66) to ultimately produce bacterial communities in older parts of the comb that are distinct from those in fresh comb (40). The increase in *Dysgonomonas* and Treponema in fresh comb, “*Candidatus* Udaeobacter” in mature comb, and *Bradyrhizobium* and Mycobacterium in old comb was the most obvious difference in bacterial communities. The fresh comb showed increased amounts of lignin units, cellulose, and hemicellulose, while it showed reduced amounts of simple sugars. Previous reports also documented the enrichment of *Dysgonomonas* (36, 50) and Treponema (40) in the fungus comb. Metagenomic analysis of Macrotermes annandalei and other termites revealed that some members of *Dysgonomonas* have the capacity to break down cellulose (67), and Treponema isolates play a key role in nitrogen fixation, reductive acetogenesis, hemicellulose degradation (68), and aromatic ring cleavage (69). Their specific enrichment in fresh comb may be associated with the lignocellulose-rich primary feces. The lignocellulolytic activity of *Termitomyces* within the comb reduces the amount of lignocellulose in mature and old comb, where the dominant bacterial taxa are also not known lignocellulose degraders. Previous reports have identified that “*Candidatus* Udaeobacter copiosus” (70) and Bradyrhizobium japonicum (71) from the soil and Mycobacterium sp. from the termites (72) can store surplus carbon as starch, fix nitrogen, and exhibit antagonistic activity in the comb, respectively, which likely represent more important bacterial functions than polysaccharide breakdown at this stage in the process.

Although mature and old combs showed similar numbers of OTUs (Fig. 6 and Table S4), mature comb occupied most bacterial diversity. It is assumed that the bacteria in mature comb are introduced from fresh comb, old comb, and surrounding soil. It is also expected that the changes in the composition might be due to the differences in conditions, comb maturation time, and lignocellulose components. These observations agree with previous work reporting a higher diversity of bacteria in the mature comb than in fresh and old combs (37, 40). These comb bacteria may work synergistically with *Termitomyces* to boost the degradation of lignocellulose components. In order to better understand the link between the digestive function and bacterial taxa in Macrotermitinae, further extensive metagenomic research can shed light on the contributions of bacteria to lignocellulose breakdown.

Because our data were derived from laboratory-reared colonies, we cannot exclude the possible effects of captivity on bacterial communities. Compared to the data of this study, bacterial communities (particularly *Alistipes*, *Dysgonomonas*, *Desulfovibrio*, *Prevotella*, Enterobacter, “*Candidatus* Tammella,” *Lactovum*, *Clostridia*, *Bacteroides*, and *Ruminococcus*) were observed in high abundances in fungus comb (35, 36) and the gut (38, 66) of free-living fungus-growing termites. However, the preponderance of these bacterial taxa in Macrotermitinae collected from widely separated locations and their presence in the symbiotic system of *O. formosanus* (this study) indicate a constant and stable relationship of these bacteria with their host. However, no systematic research has explored the comparative bacterial communities between captive and free-living fungus-growing termites.

### Conclusions.

By combining chemical analyses of the substrates and material from the fungus-growing termite *O. formosanus*, our work documented a consistent and stepwise diminution in lignocellulosic components throughout the degradation process. The digestive tract of young workers and subsequently fungus comb enables very efficient degradation of lignocellulose, resulting in the old comb material being enriched with simple carbohydrates that can be utilized by *Termitomyces* and older termites. Our finding of distinct and diverse communities of bacteria across the stages of decomposition supports the potential for bacterial roles in decomposition, but more advanced gene expression analyses are needed to clarify their possible contributions in lignocellulose degradation. Furthermore, more efforts in metabolomic research focusing on how lignocellulose components are decomposed and converted to energy and where and by what symbionts they are utilized in farming termite symbiosis are needed.

## MATERIALS AND METHODS

### Samples.

*O. formosanus* fungus-growing termite fungus combs were excised from the colonies excavated in the forest area of Hangzhou, Zhejiang, People’s Republic of China. Three mature colonies harboring king and queen were excavated. Each entire colony with fungal combs was wrapped in a bag and transported to the laboratory. Each colony was placed in a separate container (50 by 50 by 35 cm) containing soil. The rearing system was maintained in complete darkness at 27 ± 1°C and 85% relative humidity. Termites were allowed to feed on preground mulberry wood (Morus alba L.). New fungal combs were successfully built in all colonies. Fungal combs and termite guts were collected after 2 months of feeding. Each fungus comb was separately divided into three age categories (fresh, mature, and old comb) based on color and fungal growth (12). Combs with dark brown color, indicating the presence of very little fungal mycelium, were considered fresh combs. Mature combs were yellowish-brown with dense mycelia and fungal nodules. Old combs were gray with little mycelium. Termite workers were also divided into two age groups according to their abdomen color (37). Termites with blackish abdomens were considered old workers, and termites with light brown abdomens were considered young workers. The young and old workers were randomly collected and dissected to collect wood particles. The wood particles were also collected from mulberry wood as a control. Samples were stored at −80°C until analysis.

### Chemical and structural analysis.

**(i) Compositional analysis.** The contents of lignin, hemicellulose, and cellulose from original mulberry wood, fresh comb, mature comb, and old comb were analyzed by Van Soest fiber analysis (52). Cellulose was calculated from the difference between acid detergent fiber and acid detergent lignin, hemicellulose was calculated from the difference between neutral detergent fiber and acid detergent fiber, and lignin was expressed as acid detergent lignin.

Fourteen major sugar monomers were tested in this study. Xylose, ribose, rhamnose, mannuronic acid, mannose, guluronic acid, glucuronic acid, glucose, galacturonic acid, galactose, fructose, fucose, and arabinose were analyzed by using HPAEC-pulsed amperometric detection (PAD) as described in the literature (54, 73), while *N*-acetylglucosamine was determined by using HPLC-UV. For HPAEC analysis, around 5 mg dry sample was hydrolyzed in trifluoroacetic acid (1 mL 2 M) at 121°C for 120 min. After hydrolysis, a nitrogen-blowing instrument was used to dry the sample. The sample was cleaned three times by using methyl alcohol. After that, sterile water was used to dissolve the sample. The injection volume for each analysis was 20 μL. The analysis was performed on a Thermo Fisher Scientific ICS-5000 system (Sunnyvale, CA, USA). The analytical CarboPac PA10 column (250 by 4 mm, 10 μm; Dionex) was used for chromatographic separation at a 30°C column temperature with 100 mM NaOH mobile phase. The separation gradient was 2.5% for 30 min, 20% for 0.1 min, 40% for 15 min, and 2.5% for 15 min.

For HPLC analysis, approximately 0.1 g sample was ground with a grinder and then 1 mL of water was added. After being ground into a slurry, samples were subjected to ultrasound for 1 h. The supernatant (500 μL) was collected, diluted to 1 mL, and filtered. *N*-Acetylglucosamine was used as a standard. High-performance liquid chromatography was performed with a Waters 2695 (Waters, USA) and an Althna NH_2_-RP column (250 mm by 4.6 mm, 5 μm). The solvents were water (A) and acetonitrile (B), A:B ratio of 3:7. The flow rate was 0.8 mL/min, the injection volume was 10 μL, and the oven column was set at 30°C. The eluent was monitored at 195 nm.

**(ii) SEM.** The SEM (Hitachi Su8010; Japan) was used to compare the surface morphology of treated samples (fresh comb, mature comb, old comb, and young and old worker) with that of untreated mulberry wood. The young and old workers (20 to 25 individuals per sample) were dissected to collect wood particles from guts. The surfaces of young and old workers were sterilized with 70% ethanol and rinsed with phosphate-buffered saline (PBS). The guts were dissected with a sterile razor, and the contents were squeezed into the PBS. Fungus combs and wood samples were also rinsed with PBS and fixed with 2.5% glutaraldehyde in 0.1 M phosphate buffer for 10 h. Samples were then dehydrated with ethanol. After that, the 1:1 (vol/vol) mixture of ethanol and isoamyl acetate was added to each sample, incubated for 30 min, and then transferred to pure isoamyl acetate and incubated for 1 h. The samples were dehydrated in a dryer with liquid carbon dioxide. Before imaging, the samples were scattered on a double adhesive tape that stuck to an aluminum stub, coated with gold alloy. After that, the samples were scanned and photographed.

**(iii) FTIR.** The FTIR spectra were obtained using a Nicolet iS50FT-IR spectrometer (Thermo Scientific, USA) to compare the structural changes in lignocellulosic components across different stages of decomposition. The samples were dried at 40°C and milled to a fine powder (size less than 250 μm). Then, the sample (approximately 2 mg) was mixed with potassium bromide (KBr) and scanned. The spectra were collected over a wavenumber range of 400 cm^−1^ to 4,000 cm^−1^ at a resolution of 4 cm^−1^ (55).

**(iv) XRD.** The crystallinity of samples was determined by X-ray diffraction (XRD) analysis using an X-ray diffractometer (Bruker D8; Germany). The analysis was performed as described previously by Xu et al. (56). The operating conditions were 40-kV voltage and 40-mA current with Cu Kα radiations as an X-ray source. The scanning range was 5 to 40° at the 2θ angle. The crystallinity index (CrI) was calculated using the equation CrI = (*I*_Cr_ − *I*_Am_)/*I*_Cr_ × 100, where *I*_Cr_ represents the maximum diffraction intensity at peak position 2θ = 22° and *I*_Am_ is the intensity at 2θ = 18° (74, 75).

**(v) Py-GC-MS.** The compositions of lignin monomers in *Morus alba* and different parts of fungus comb samples were determined by using pyrolysis-gas chromatography/mass-spectrometry (Py-GC-MS). The pyrolysis processes were performed as described in previous reports (1, 27). Briefly, the sample and 3,5-dimethoxyphenol (internal standard) were placed into the quartz tube. Each quartz sample tube contained approximately 1 mg sample and 0.005 mg internal standard. Samples were pyrolyzed using a pyrolysis autosampler (EGA/PY-3030D; Frontier Laboratories Ltd., Japan) coupled with an Agilent GC-MS system (GC/MSD 7890B-5977A; Agilent Technologies, USA). The samples were pretreated at a temperature of 150°C for 3 min and then pyrolyzed at 610°C for 30 s. Chromatographic separation was achieved using an HP-5MS nonpolar capillary column (30 m in length, 0.25 mm in inside diameter [i.d.], and 0.25 μm in film thickness) with helium (He) as a carrier gas (1 mL/min). The oven temperature of GC-MS was 40°C for 1 min to 280°C for 15 min at the rate of 6°C per min. The GC-MS interface and the pyrolysis interface were kept at 280°C and 210°C, respectively. The mass spectrometer was operated in electron impact (EI) ionization mode (70 eV) at an ion-source temperature of 230°C. The compounds were determined by interpretation of their mass spectra and comparison with the National Institute of Standards and Technology (NIST) electronic library.

### Amplicon sequencing of bacterial communities.

The surfaces of young and old worker termites were sterilized with 70% ethanol and rinsed with PBS. The guts (20 to 25 individuals per sample) were dissected using sterile forceps. Samples were homogenized using a sterile glass rod. Then the homogenates were transferred into tubes for DNA extraction. The combs (fresh, mature, and old comb) were ground to a fine powder using a pestle and mortar. The samples (0.5 g of each sample) were homogenized separately and used for DNA extraction.

DNA was extracted from different samples using an Omega soil DNA kit (M5635-02) (Omega Bio-Tek, Norcross, GA, USA) according to the manufacturer’s instructions. The V3-V4 regions of the bacterial 16S rRNA gene were amplified with forward 341F 5′-CCTAYGGGRBGCASCAG-3′ and reverse 806R 5′-GGACTACHVNNGGGTATCTAAT-3′ primers (36, 38, 40). Sample-specific 7-bp barcodes were incorporated into the primers for multiplex sequencing. The PCR amplification reaction mixture was prepared in a 25-μL volume containing 2 μL deoxynucleoside triphosphate (dNTP) (2.5 mM), 1 μL DNA, 1 μL of each primer (10 μM), 14.75 μL double-distilled water (ddH_2_O), and 0.25 μL fast *Pfu* DNA polymerase (New England Biolabs [NEB], USA). The PCR was performed at 98°C for 5 min, 25 cycles of 98°C for 30 s, 55°C for 30 s, and 72°C for 45 s, and 72°C for 5 min. Agarose gel electrophoresis was used to visualize PCR products. Then, a Quant-iT PicoGreen double-stranded DNA (dsDNA) kit (Invitrogen, CA) was used to quantify the DNA concentrations. The samples were sequenced on an Illumina MiSeq sequencer by Personalbio Biotech (Shanghai, China).

### Statistical analyses.

Comparisons of data regarding plant components at different stages of decomposition were made through one-way analysis of variance (ANOVA) using SPSS, v.22.0 (SPSS Inc., Chicago, IL) at α = 0.05. Microbiome composition analyses were performed with QIIME2 2019.4 (76, 77) with slight modification according to the official tutorials (https://docs.qiime2.org/2019.4/tutorials/). Briefly, raw sequence data were demultiplexed using the demux plugin following by primer cutting with the cutadapt plugin (78). Sequences were then merged, quality filtered, and dereplicated using functions of fastq_mergepairs, fastq_filter, and derep fullength in the Vsearch plugin. All the unique sequences were then clustered at 98% (via cluster size) followed by chimera removal (via Uchime *de novo*) (79). Nonchimera sequences were clustered at 97% similarity using the UCLUST algorithm to generate operational taxonomic unit (OTU) representative sequences (80). Nonsingleton amplicon sequence variants (OTUs) were aligned with mafft (81) and used to construct a phylogeny with fasttree2 (82). Taxonomy was assigned to OTUs using the classify-sklearn naive Bayes taxonomy classifier in the feature-classifier plugin (83) against the SILVA Release 138 database (http://www.arb-silva.de) (84) using mothur v.1.44.3 (85).

Alpha diversity index or indices including Chao1, observed species, Simpson, Shannon, Good’s coverage, and Pielou’s evenness were calculated using QIIME2 (36) and visualized as box plots. The differences in α diversity were evaluated using the Kruskal-Wallis test (*H*-test) and Dunn’s *post hoc* test. Beta diversity analysis was performed to investigate the structural variation of microbial communities across samples using Jaccard metrics (86) and Bray-Curtis metrics (87) and visualized via principal-coordinate analysis (PCoA). Venn diagrams, principal-coordinate analysis, and heatmaps were used to map and compare the bacterial relative abundances at different levels using R Studio version 3.6.0 (http://www.r-project.org) (88). The linear discriminant analysis effect size (LEfSe) algorithm was used to determine different abundances of bacterial communities at different stages of decomposition using the default parameters (89).

### Data availability.

The sequences from this study have been deposited at the National Center for Biotechnology Information (NCBI) in the Sequence Read Archive (SRA) under BioProject accession no. PRJNA836728, which is publicly available at http://www.ncbi.nlm.nih.gov.
