# Supplementary material for: Tripartite Symbiotic Digestion of Lignocellulose in the Digestive System of a Fungus-Growing Termite
Source: Microbiol Spectr. 2022 Oct 17;10(6):e01234-22. doi: 10.1128/spectrum.01234-22 (PMC9769757; doi:10.1128/spectrum.01234-22)
Supplement: Supplemental file 1 — Tables S1 to S7 and Fig. S1 to S3. Download spectrum.01234-22-s0001.pdf, PDF file, 1.3 MB [file spectrum.01234-22-s0001.pdf]

Supplementary Table S1. Lignocellulose composition of original mulberry, fresh comb, mature comb, and old comb.

**Polysaccharides**	**Mulberry wood**	**Fresh comb**	**Mature comb**	**Old comb**	**LSD**
Lignin (mg/g)	256.6 ± 9.24	208.2 ± 4.41 (-18.9%)	110.6 ± 1.87 (-56.9%)	094.9 ± 2.09 (-63.0%)	<0.001
Cellulose (mg/g)	513.3 ± 8.23	456.5 ± 8.60 (-11.1%)	303.0 ± 7.39 (-41.0%)	176.9 ± 5.86 (-65.5%)	<0.001
Hemicellulose (mg/g)	152.5 ± 2.50	129.6 ± 1.86 (-15.0%)	102.9 ± 1.21 (-32.5%)	071.0 ± 1.47 (-53.4%)	<0.001
Sugar monomers (µg/mg)*					
Fucose	NF	NF	NF	0.013 ± 0.001	<0.001
Rhamnose	NF	0.007 ± 0.00	0.006 ± 0.006	0.009 ± 0.000	<0.001
Arabinose	0.037 ± 0.001	0.025 ± 0.001 (-32.4%)	0.029 ± 0.000 (-21.6%)	0.047 ± 0.002 (27.0%)	<0.001
Galactose	0.108 ± 0.002	0.038 ± 0.001 (-64.8%)	0.025 ± 0.000 (-76.9%)	0.073 ± 0.003 (-32.4%)	<0.001
Glucose	1.335 ± 0.011	1.633 ± 0.004 (22.3%)	1.715 ± 0.048 (28.5%)	3.258 ± 0.050 (144.0%)	<0.001
Xylose	0.030 ± 0.001	0.320 ± 0.003 (966.7%)	0.184 ± 0.011 (513.3%)	0.617 ± 0.012 (1956.7%)	<0.001
Mannose	0.112 ± 0.002	0.058 ± 0.002 (-48.2%)	0.066 ± 0.001 (-41.1%)	0.090 ± 0.003 (-19.6%)	<0.001
Fructose	2.109 ± 0.005	0.041 ± 0.002 (-98.1%)	0.040 ± 0.002 (-98.1%)	0.149 ± 0.004 (-92.9%)	<0.001
Ribose	NF	0.086 ± 0.001	0.063 ± 0.002	0.062 ± 0.002	<0.001
Galacturonic acid	0.029 ± 0.002	0.034 ± 0.002 (17.2%)	0.033 ± 0.001 (13.8%)	0.105 ± 0.001 (262.1%)	<0.001
Guluronic acid	NF	NF	NF	NF	NF
Glucuronic acid	0.027 ± 0.002	0.034 ± 0.011 (25.9%)	0.144 ± 0.021 (433.3%)	0.043 ± 0.012 (59.3%)	<0.001
Mannuronic acid	NF	NF	NF	NF	NF
N-Acetylglucosamine	NF	0.046 ± 0.000	0.007 ± 0.000	0.059 ± 0.000	<0.001

*The amount of each monomer is expressed as µg/mg of the dry sample weight. Values are expressed as mean determined of three replicates ± SE (Percentage change). NF= Not found.

Supplementary Table S2. Common absorption FTIR bands observed in spectra of mulberry wood and all stages of comb, accompanied by corresponding bond vibrations and functional groups.

Sr. #	Wavenumber (cm^{-1})	Vibration	Functional group and/or compound	References
1	1735	C=O stretching	Aldehydes, carboxylic acids, and ketones associated with hemicellulose	(1-5)
2	1624	Absorbed O=H	Water	(6)
		Conjugated C-O stretching	Carbohydrates	(6)
		Carbonyl C=O stretching	Primary and secondary amides	(4, 5, 7)
		NH2 deformation	Primary amides	(5)
		Aromatic ring C=C stretching	Aromatic compounds	(5)
3	1509	C=C stretching	Lignin	(1-4)
4	1458	Asymmetric C-H bending	Aliphatic methylene	(5)
5	1425	Symmetric C-H bending	Lignin and polysaccharides	(1, 2, 4)
		C-O stretching/O-H deformation	Phenolic compounds	(8)
6	1376	Symmetric C-H bending	Cellulose and hemicellulose	(1, 2)
7	1321	C-N stretching	Secondary amides	(5)
		C-O stretching	Syringyl ring in lignin	(4)
8	1247	C-O stretching	Syringyl ring in lignin	(2, 6)
			Guaiacyl ring in lignin	(1, 3, 7)
9	1204	O-H bending	Cellulose and hemicellulose	(1)

10	1162	C-O-C stretching	Cellulose and hemicellulose	(3-5, 9)
11	1113	C-O stretching	Cellulose	(4, 5, 9)
12	1051	C-O stretching, deformation	Cellulose and polysaccharides	(7)
13	1036	C-H deformation	Lignin	(4, 7)
		C-O, C=C, C-C-O stretching	Lignin, cellulose, and hemicellulose	(1, 3)
14	896	C-H deformation	β-pyranose compounds	(5)
			Cellulose	(3, 4, 7, 9)
15	834	C-H bending	Lignin	(10)
16	780	C-H bending	Lignin	(11)

Supplementary Table S3. Chemical composition of mulberry wood, fresh, mature, and old comb in comparison with other reports on fungus-growing termites.

Sample	Termite species	Food	Lignin	Cellulose	Hemicellulose	Fucose	Rhamnose	Arabinose	Galactose	Glucose	Xylose	Mannose	Fructose	Ribose	Galacturonic acid	Guluronic acid	Glucuronic acid	Mannuronic acid	N-Acetyl-glucosamine	References
Mulberry wood	*Odontotermes formosanus*		25.66	51.33	15.25	0	0	0.0037	0.0108	0.1335	0.003	0.0112	0.2109	0	0.0029	0	0.0027	0	0	This study
Decaying wood	*Macrotermes natalensis*		9.27-20.61	27.12-28.19		0.007-0.040		0.304-0.949	0.959-1.165	2.525-4.454	3.345-13.12	0.514			0.621-1.069		0.126-0.170			12
Bark	*M. natalensis*		8.56-14.86	35.90-43.88		0-0.004		0.076-0.558	0.298-1.194	2.867-3.797	3.055-4.991	0.303			0.072-0.426		0.099-0.117			12
Dry wood	*Odontotermes* sp.		7.94-13.99	9.695-35.76		0.024-0.083		0.269-5.334	0.903-3.146	2.274-4.035	4.081-12.89	0.365-1.035			0.997-3.097		0.123-0.442			12
	O. badius		10.71-12.72	16.37-28.01		0.051-0.075		0.644-1.086	0.968-1.203	2.56-3.246	10.94-13.78	0.588-0.687			0.777-1.039		0.144-0.177			12
	M. natalensis		8.50-16.23	23.54-40.53		0-0.009		0.2622-1.030	0.961-1.677	1.933-5.147	1.862-15.22	0.570-1.435			0.129-1.711		0.122-0.252			12
Cow dung	*Odontotermes* sp.		13.93	26.1		0.007		1.336	0.716	1.633	5.912	0.359			0.13		0.174			12
	O. badius		13.91	16.05		0.006		1.309	0.701	1.581	5.764	0.301			0.129		0.163			12
	M. natalensis		11.27-13.30	14.11-26.08		0.006		0.889-1.806	0.558-0.903	1.124-1.954	3.741-4.486	0.173-0.380			0.088-0.108		0.091-0.175			12
Popular wood	*O. formosanus*		22					1	1	50	22	2					2			13
Fresh comb	*O. formosanus*	Mulberry	20.82	45.65	12.96	0	0.0007	0.0025	0.0038	0.1633	0.032	0.0058	0.0041	0.009	0.0034	0	0.0034	0	0.0046	This study
	Odontotermes sp.	unknown	10.73-14.71	11.73-13.44		0.079-0.174		0.8705-2.319	0.711-1.899	2.338-5.139	3.000-6.241	0.669-1.242			0.334-0.833		0.243-0.329			12
	O. badius	unknown	13.73-14.93	12.77-16.81		0.068-0.101		1.062-1.62	0.594-1.065	1.977-2.593	2.867-4.526	0.534-0.634			0.678-0.783		0.238-0.327			12
	M. natalensis	unknown	9.70-14.44	11.15-16.01		0.094-0.157		0.9845-3.48	0.671-2.368	3.457-7.019	3.844-13.83	0.487-1.079			0.148-0.698		0.200-0.314			12
	O. formosanus	Popular	13						2	40	12	1								13

Mature comb	*O. formosanus*	Mulberry	11.06	30.3	10.29	0	0.0006	0.0029	0.0025	0.1715	0.018	0.0066	0.004	0.006	0.0033	0	0.0144	0	0.0007	This study
	O. formosanus	Popular	7						2	38	10	1								13
Old comb	*O. formosanus*	Mulberry	9.49	17.69	7.1	0.001	0.0009	0.0047	0.0073	0.3258	0.062	0.009	0.0149	0.006	0.0105	0	0.0043	0	0.0059	This study
	Odontotermes sp.	unknown	8.020-14.06	8.128-11.83		0.096-0.174		0.9336-1.347	0.948-1.236	2.539-4.034	3.147-4.91	0.594-1.125			0.434-0.743		0.184-0.243			12
	O. badius	unknown	8.432-14.06	4.393-10.28		0.105-0.129		1.028-1.616	0.788-1.047	2.863-3.252	2.507-4.425	0.607-0.905			0.841-1.196		0.238-0.332			12
	M. natalensis	unknown	7.625-14.91	5.343-23.48		0.107-0.136		0.9845-2.373	0.671-1.773	3.457-6.549	6.856-9.236	0.487-0.908			0.156-0.698		0.153-0.239			12
	O. formosanus	Popular	5						4	41	5	1								13

Supplementary Table S4. Estimated bacterial community diversity, richness and evenness in fungus-growing termite guts and three comb categories.

Sample	Number of Sequences	Chao1	Goods_ coverage	Observed_ species	Pielou_e	Shannon	Simpson
FC1	103740	1301	0.9929	1070	0.6089	6.127	0.9506
FC2	135690	2333	0.9862	1683	0.5217	5.591	0.8337
FC3	148217	1927	0.9893	1329	0.6441	6.683	0.9630
MC1	135532	2893	0.9888	2547	0.7449	8.428	0.9793
MC2	144627	2245	0.9899	1879	0.6195	6.737	0.9458
MC3	136321	2695	0.9875	2249	0.6541	7.283	0.9529
OC1	140547	2420	0.9902	2102	0.6173	6.814	0.9140
OC2	134643	2860	0.9859	2413	0.5137	5.773	0.8419
OC3	141146	2732	0.9862	2255	0.5749	6.403	0.8928
OW1	118689	2198	0.9865	1558	0.6309	6.690	0.9725
OW2	113889	1770	0.9899	1316	0.6341	6.571	0.9643
OW3	115121	2176	0.9873	1462	0.6585	6.923	0.9742
YW1	127904	1615	0.9922	1325	0.6933	7.191	0.9769
YW2	132511	1515	0.9923	1246	0.6480	6.664	0.9655
YW3	125874	1515	0.9920	1245	0.6151	6.325	0.9531

Supplementary Table S5. Relative abundance of bacterial phyla across different stages in the decomposition process.

Phylum	Fresh fungus comb (%)				Mature fungus comb (%)				Old fungus comb (%)				Old worker (%)				Young worker (%)			
	**FC1**	**FC2**	**FC3**	**Mean**	**MC1**	**MC2**	**MC3**	**Mean**	**OC1**	**OC2**	**OC3**	**Mean**	**OW1**	**OW2**	**OW3**	**Mean**	**YW1**	**YW2**	**YW3**	**Mean**
Bacteroidota	54.2	27.95	57.39	46.51	5.96	3.33	25.13	11.47	6.28	2.24	15.02	7.85	37.77	38.9	50.28	42.32	40.88	39.73	45.92	42.18
Firmicutes	17.97	56.85	16.69	30.50	30.35	67.17	38.15	45.22	23.74	5.28	29.34	19.45	26.5	20.81	21.07	22.79	20.88	36.49	16.5	24.62
Proteobacteria	0.83	3.63	2	2.15	29.6	13.51	24.09	22.40	46.59	53.23	41.74	47.19	9.37	20.14	2.78	10.76	5.66	0.38	4.48	3.51
Actinobacteriota	3.73	1.95	1.61	2.43	6.25	2.9	3.39	4.18	4.72	23.87	3.59	10.73	9.67	6.22	5.96	7.28	6.4	3.42	3.85	4.56
Synergistota	1.31	0.59	1.7	1.20	0.02	0.02	0.01	0.02	0.02	0	0	0.01	3.01	3.95	6.83	4.60	13.87	9.16	17.9	13.64
Spirochaetota	15.69	3.45	11.36	10.17	0.14	0	0.04	0.06	0	0.02	0.06	0.03	0.07	0.65	0.15	0.29	0.55	0.77	0.25	0.52
Planctomycetota	3.58	0.48	2.71	2.26	0.49	0.17	0.06	0.24	0.19	0.45	0.09	0.24	4.42	3.24	4.26	3.97	4.87	3.22	3.35	3.81
Acidobacteriota	0.12	0.68	0.09	0.30	9.14	4.03	2.68	5.28	5.78	4.85	3.07	4.57	0.52	0.15	0.38	0.35	0.01	0.01	0.01	0.01
Desulfobacterota	0.49	0.5	0.77	0.59	0.15	0.23	0.41	0.26	0.33	0.09	0.18	0.20	5.03	3.71	4.07	4.27	3.76	3.68	4.37	3.94
Verrucomicrobiota	0.1	0.47	0.06	0.21	6.7	3.05	1.09	3.61	5.5	2.48	1.9	3.29	0.46	0.06	0.19	0.24	0	0	0.02	0.01
Patescibacteria	0.99	1.01	2.96	1.65	1.67	0.53	0.51	0.90	0.87	0.94	0.51	0.77	0.85	0.33	1.56	0.91	1.26	1.29	2.11	1.55
Myxococcota	0.08	0.29	0.07	0.15	2.76	1.38	0.73	1.62	1.59	1.48	0.8	1.29	0.14	0.05	0.06	0.08	0	0.01	0.03	0.01
Chloroflexi	0.04	0.17	0.04	0.08	2.51	0.99	0.67	1.39	1.37	1.69	0.8	1.29	0.17	0.04	0.1	0.10	0.01	0	0	0.00
Rs-K70_termite_group	0.13	0.12	0.13	0.13	0	0	0	0.00	0	0	0	0.00	1.33	1.24	1.34	1.30	0.87	0.67	0.76	0.77
Gemmatimonadota	0.01	0.1	0.01	0.04	1.15	0.64	0.41	0.73	0.95	0.94	0.47	0.79	0.14	0.01	0.04	0.06	0	0	0	0.00
Campilobacterota	0.05	0.13	0.11	0.10	0	0.03	0.51	0.18	0.03	0	1.05	0.36	0.23	0.34	0.68	0.42	0.85	0.47	0.29	0.54
Elusimicrobiota	0.01	0.18	0.52	0.24	0.38	0.1	0.16	0.21	0.19	0.14	0.09	0.14	0.02	0.01	0.09	0.04	0.02	0.04	0.03	0.03
Cyanobacteria	0	0.08	0.02	0.03	0.12	0.43	0.91	0.49	0.15	0.15	0.05	0.12	0.02	0.01	0.01	0.01	0.01	0.01	0	0.01
Methylomirabilota	0.01	0.05	0.01	0.02	0.35	0.3	0.14	0.26	0.3	0.34	0.27	0.30	0.03	0	0.03	0.02	0	0	0	0.00
Dependentiae	0	0.06	0.01	0.02	0.54	0.17	0.21	0.31	0.28	0.23	0.19	0.23	0.02	0.01	0.01	0.01	0	0	0	0.00
Others	0.66	1.27	1.77	1.23	1.71	1.01	0.72	1.15	1.13	1.58	0.76	1.16	0.23	0.12	0.13	0.16	0.1	0.64	0.11	0.28

Supplementary Table S6. Relative abundance of bacteria families throughout the decomposition process.

Family	Fresh fungus comb (%)				Mature fungus comb (%)				Old fungus comb (%)				Old worker (%)				Young worker (%)			
	**FC1**	**FC2**	**FC3**	**Mean**	**MC1**	**MC2**	**MC3**	**Mean**	**OC1**	**OC2**	**OC3**	**Mean**	**OW1**	**OW2**	**OW3**	**Mean**	**YW1**	**YW2**	**YW3**	**Mean**
Rikenellaceae	29.82	11.31	31.42	24.18	0.47	0.08	0.82	0.46	0.17	0.06	0.33	0.19	27.31	24.54	34.33	28.73	24.84	24.9	28.54	26.09
Lachnospiraceae	10.66	44.25	5.41	20.11	14.04	28.17	11.45	17.89	6.15	0.73	4.72	3.87	1.36	1.02	0.6	0.99	0.55	0.39	0.56	0.5
Xanthobacteraceae	0.1	0.47	0.41	0.33	7.76	2.88	15.23	8.62	4.74	36.76	32.87	24.79	3.44	0.55	0.56	1.52	0.01	0.01	0	0.01
Tannerellaceae	6.84	2.4	9.26	6.17	0.05	0.24	0.38	0.22	0.19	0.05	0.09	0.11	5.29	6.7	9.22	7.07	11.92	13.1	15.48	13.5
Enterobacteriaceae	0.15	0.99	0.04	0.39	1.14	1.76	1	1.3	26.95	1.65	1.9	10.17	4.72	18.85	1.32	8.3	4.43	0.12	4.06	2.87
Ruminococcaceae	2.69	0.85	2.49	2.01	0.36	0.28	3.51	1.38	0.39	0.11	1.93	0.81	9.47	7.48	10.55	9.17	10.51	6.02	7.8	8.11
Synergistaceae	1.31	0.59	1.7	1.2	0.02	0.02	0.01	0.02	0.02	0	0	0.01	3.01	3.95	6.83	4.6	13.87	9.16	17.9	13.64
Bacillaceae	0.2	0.79	0.12	0.37	11.61	7.01	10.36	9.66	1.21	1.61	1.21	1.34	0.04	0	0	0.01	0.02	15.22	0.14	5.13
Dysgonomonadaceae	11.75	9.7	10.16	10.54	0.05	0.02	0.11	0.06	0	0.04	0.01	0.02	2.58	5.64	5.27	4.5	1.53	0.44	0.65	0.87
Spirochaetaceae	15.69	3.45	11.36	10.17	0.13	0	0.04	0.06	0	0.02	0.05	0.02	0.07	0.65	0.15	0.29	0.55	0.77	0.25	0.52
Lactobacillaceae	0.02	0.56	0.08	0.22	0.35	2.39	1	1.25	10.69	0.2	13.58	8.16	0.25	0.37	0.04	0.22	0.62	0.13	0.09	0.28
vadinHA49	3.55	0.47	2.7	2.24	0.02	0	0.02	0.01	0	0.01	0	0	4.39	3.23	4.19	3.94	4.77	3.22	3.29	3.76
Desulfovibrionaceae	0.47	0.45	0.75	0.56	0.02	0.12	0.32	0.15	0.19	0.01	0.1	0.1	4.98	3.68	3.99	4.22	3.66	3.61	4.31	3.86
Prevotellaceae	0	0	0.13	0.04	0.02	0.09	16.8	5.64	0.12	0.08	6.83	2.34	0.59	0.68	0.04	0.44	0.03	0.09	0.16	0.09
Hungateiclostridiaceae	0.17	3.58	0.39	1.38	0.04	18.55	0.03	6.21	0	1.16	0	0.39	0.06	0.03	0.16	0.08	0.11	0.22	0.12	0.15
Erysipelotrichaceae	0.08	0.07	0.01	0.05	0.29	0.68	0.07	0.35	1.11	0.33	0.05	0.5	8.3	4.55	3.36	5.4	0.82	0.84	1.3	0.99
Mycobacteriaceae	0.06	0.09	0.2	0.12	0.41	0.16	0.24	0.27	0.2	19.77	0.12	6.7	0.02	0.06	0.01	0.03	0	0	0	0
Propionibacteriaceae	2.9	0.72	0.68	1.43	0.17	0.06	0.12	0.12	0.07	0.08	0.02	0.06	5.59	3.4	2.66	3.88	0.74	1.24	1.63	1.2
Chthoniobacteraceae	0.06	0.33	0.04	0.14	5.57	2.46	0.91	2.98	3.77	2	1.54	2.44	0.38	0.05	0.15	0.19	0	0	0	0
Muribaculaceae	0	0.02	0.05	0.02	0.37	1	4.83	2.07	2.49	0.06	4.92	2.49	0.33	0.31	0.03	0.22	0.03	0.04	0.09	0.05
Others	13.47	18.88	22.6	18.32	57.09	34.03	32.74	41.29	41.52	35.27	29.7	35.5	17.81	14.26	16.52	16.2	20.98	20.47	13.61	18.35

Supplementary Table S7. Relative abundance of bacterial genera across the different stages of decomposition.

Genus	Fresh fungus comb (%)				Mature fungus comb (%)				Old fungus comb (%)				Old worker (%)				Young worker (%)			
	FC1	FC2	FC3	Mean	MC1	MC2	MC3	Mean	OC1	OC2	OC3	Mean	OW1	OW2	OW3	Mean	YW1	YW2	YW3	Mean
Alistipes	27.66	9.65	28.88	22.06	0.32	0.08	0.75	0.38	0.15	0.05	0.25	0.15	27.25	24.38	33.97	28.53	23.67	24.55	28.32	25.51
Bradyrhizobium	0.03	0.14	0.34	0.17	1.82	0.82	13.36	5.33	1.28	34.33	30.9	22.17	3.08	0.46	0.4	1.31	0.01	0	0	0
Parabacteroides	4.76	1.4	6.67	4.28	0.05	0.24	0.29	0.19	0.19	0.04	0.09	0.11	3.24	2.86	4.74	3.61	6.29	9.61	12.4	9.43
Dysgonomonas	11.71	9.58	10.13	10.47	0.05	0.02	0.11	0.06	0	0.04	0.01	0.02	2.52	5.61	5.23	4.45	1.44	0.42	0.6	0.82
Bacillus	0.19	0.78	0.12	0.36	11.39	6.94	10.29	9.54	1.16	1.55	1.16	1.29	0.04	0	0	0.01	0	10.21	0.05	3.42
Escherichia-Shigella	0.14	0.65	0.03	0.27	0.79	1.66	0.94	1.13	26.67	1.6	1.64	9.97	0.06	0.02	0.06	0.05	0.08	0	0.01	0.03
Treponema	15.41	3.21	10.97	9.86	0.1	0	0.04	0.05	0	0	0.05	0.02	0.07	0.59	0.15	0.27	0.51	0.75	0.23	0.5
Lactobacillus	0.02	0.56	0.08	0.22	0.35	2.39	1	1.25	10.67	0.19	13.58	8.15	0.25	0.37	0.04	0.22	0.62	0.09	0.09	0.27
vadinHA49	3.55	0.47	2.7	2.24	0.02	0	0.02	0.01	0	0.01	0	0	4.39	3.23	4.19	3.94	4.77	3.22	3.29	3.76
Fusicatenibacter	0.16	0.85	0	0.34	0.01	18.16	0.32	6.16	3.87	0.37	0.02	1.42	0	0	0	0	0	0	0	0
Desulfovibrio	0.35	0.32	0.62	0.43	0.01	0.03	0.04	0.03	0.18	0	0.04	0.07	4.71	3.45	3.61	3.92	3.14	3.09	3.83	3.35
Mycobacterium	0.06	0.09	0.2	0.12	0.41	0.16	0.24	0.27	0.2	19.77	0.12	6.7	0.02	0.06	0.01	0.03	0	0	0	0
Prevotella	0	0	0.09	0.03	0.02	0.07	14.91	5	0.07	0.04	5.15	1.75	0.1	0.2	0.01	0.1	0.01	0.07	0.13	0.07
Breznakia	0.05	0.04	0.01	0.03	0	0	0	0	0	0	0	0	7.79	4.4	3.16	5.12	0.6	0.76	1.21	0.86
Enterobacter	0.01	0.31	0.01	0.11	0.31	0.08	0.05	0.15	0.26	0.04	0.21	0.17	4.31	4.55	1.1	3.32	1.95	0.12	4.01	2.03
Candidatus_Udaeobacter	0.06	0.33	0.04	0.14	5.47	2.45	0.91	2.94	3.67	1.97	1.52	2.39	0.38	0.05	0.15	0.19	0	0	0	0
Kosakonia	0	0	0	0	0	0	0	0	0	0	0	0	0	13.85	0	4.62	2.28	0	0	0.76
Aestuariimicrobium	2.79	0.66	0.53	1.33	0.05	0	0	0.02	0	0	0	0	3.8	2.48	2.19	2.82	0.58	1	1.29	0.96
Muribaculaceae	0	0.02	0.05	0.02	0.37	1	4.83	2.07	2.49	0.06	4.92	2.49	0.33	0.31	0.03	0.22	0.03	0.04	0.09	0.05
Candidatus_Vestibaculum	1.09	0.81	1.65	1.18	0	0	0.08	0.03	0	0	0	0	0.64	1.08	1.31	1.01	3.33	1.81	1.45	2.2
Bacteroides	1.67	0.6	1.75	1.34	0.68	0.86	0.78	0.77	1.29	0.29	1.63	1.07	0.75	0.42	0.76	0.64	1.07	0.3	0.31	0.56
Pseudobacteroides	0	1.67	0.01	0.56	0	9.51	0	3.17	0	1.03	0	0.34	0	0	0	0	0	0	0	0
Candidatus_Tammella	0.17	0.08	0.2	0.15	0.01	0	0	0	0	0	0	0	0.93	0.9	1.31	1.05	2.34	2.07	4.04	2.82
Lactovum	1.54	1.59	0.59	1.24	0.01	0	0.01	0.01	0	0	0	0	1.45	1.25	2.17	1.62	0.87	0.64	1.59	1.03

Clostridia_UCG-014	0.01	0.22	2.78	1	0.09	0.34	2.6	1.01	0.11	0.01	1.6	0.57	0.05	0.06	0.1	0.07	0.04	0	0.03	0.02
Ruminiclostridium	0	1.45	0	0.48	0.03	6.38	0.01	2.14	0	0.06	0	0.02	0	0	0	0	0	0	0	0
M2PB4-61_termite_group	1.96	1.55	2.4	1.97	0.06	0	0.04	0.03	0.02	0.01	0	0.01	0.03	0.1	0.19	0.11	0.92	0.26	0.15	0.44
Christensenellaceae_R-7_group	0.16	0.28	0.38	0.27	0.02	0.25	0.12	0.13	0.02	0	0.11	0.04	1.1	0.87	0.93	0.97	1	0.78	1.09	0.96
Clostridia_vadinBB60_group	0.58	1.13	2.25	1.32	0.01	0.71	1.9	0.87	0.02	0.01	0.03	0.02	0.02	0.02	0.01	0.02	0.07	0.03	0.06	0.05
Rs-K70_termite_group	0.13	0.12	0.13	0.13	0	0	0	0	0	0	0	0	1.33	1.24	1.34	1.3	0.87	0.67	0.76	0.77
Tyzzerella	0.78	1.84	1.94	1.52	0.35	0.05	0.04	0.15	0.01	0.03	0	0.01	0.42	0.26	0.14	0.27	0.25	0.19	0.22	0.22
Candidatus_Saccharimonas	0.31	0.22	0.97	0.5	0	0	0.1	0.03	0	0	0.08	0.03	0.45	0.2	0.95	0.53	0.87	0.84	1.42	1.04
Tepidimicrobium	0	0	0	0	0.12	0	0	0.04	0.02	0.01	0	0.01	0	0	0	0	0	5.91	0.06	1.99
Saccharimonadales	0.61	0.25	1.41	0.76	0.52	0.12	0.12	0.25	0.29	0.21	0.08	0.19	0.19	0.06	0.21	0.15	0.22	0.26	0.38	0.29
Sinibacillus	0	0	0	0	0.13	0.01	0	0.05	0.03	0.02	0.02	0.02	0	0	0	0	0	4.68	0.02	1.57
Raoultibacter	0.06	0.06	0.03	0.05	0	0	0	0	0	0	0	0	1.44	0.79	0.86	1.03	0.28	0.5	0.53	0.44
BCf9-17_termite_group	1.41	1.46	1.35	1.41	0.01	0	0.03	0.01	0	0	0	0	0	0	0.01	0	0.04	0.01	0.01	0.02
Subgroup_2	0.02	0.06	0.02	0.03	1.16	0.49	0.4	0.68	0.81	0.68	0.4	0.63	0.07	0.03	0.07	0.06	0	0	0	0
Ruminococcus	0	0	0.03	0.01	0.02	0.02	2.79	0.94	0	0.02	1.1	0.37	0.09	0.09	0.01	0.06	0.01	0.02	0.02	0.02
Corynebacterium	0	0.01	0	0	0.12	0.01	0.09	0.07	0.01	0.05	0.05	0.04	0	0	0	0	3.47	0.25	0	1.24
Candidatus_Solibacter	0.02	0.09	0.01	0.04	1.13	0.49	0.4	0.67	0.7	0.6	0.38	0.56	0.05	0.01	0.07	0.04	0	0	0	0
Allorhizobium-Neorhizobium-Pararhizobium-Rhizobium	0.06	0.12	0.04	0.07	0.69	0.64	0.57	0.63	0.85	0.45	0.43	0.58	0.03	0.01	0.03	0.02	0.01	0.01	0.01	0.01
Lachnospiraceae_NK4A136_group	0.08	0.01	0.05	0.05	0.12	0.16	1.69	0.66	0.06	0.02	1.42	0.5	0.07	0.06	0.04	0.06	0.02	0.01	0.08	0.04
Haliangium	0.03	0.08	0.02	0.04	1.25	0.47	0.26	0.66	0.62	0.57	0.38	0.52	0.06	0.02	0.02	0.03	0	0	0	0
Bryobacter	0.02	0.1	0.01	0.04	1.12	0.43	0.26	0.6	0.52	0.57	0.4	0.5	0.06	0.02	0.03	0.04	0	0	0	0
Candidatus_Soleaferrea	0.29	0.19	0.78	0.42	0.05	0.01	0	0.02	0.01	0.01	0	0.01	0.44	0.36	0.27	0.36	0.27	0.21	0.22	0.23
Paenibacillus	0.03	0.11	0.01	0.05	1.29	0.95	0.37	0.87	0.11	0.09	0.07	0.09	0.01	0	0	0	0	0	0.01	0
Christensenellaceae	0.2	0.08	0.19	0.16	0	0	0.01	0	0	0	0	0	0.47	0.36	0.34	0.39	0.48	0.4	0.37	0.42
KD4-96	0.01	0.06	0.02	0.03	0.88	0.3	0.17	0.45	0.48	0.44	0.28	0.4	0.08	0.02	0.04	0.05	0	0	0	0
Rs-E47_termite_group	1.06	0.55	0.97	0.86	0.01	0	0.01	0.01	0	0	0	0	0.01	0.02	0.02	0.02	0.04	0.05	0.01	0.03
Others	20.76	56.14	18.55	31.82	68.53	43.69	39.07	50.43	43.19	34.73	31.84	36.59	27.43	24.85	29.71	27.33	37.54	26.16	31.63	31.78

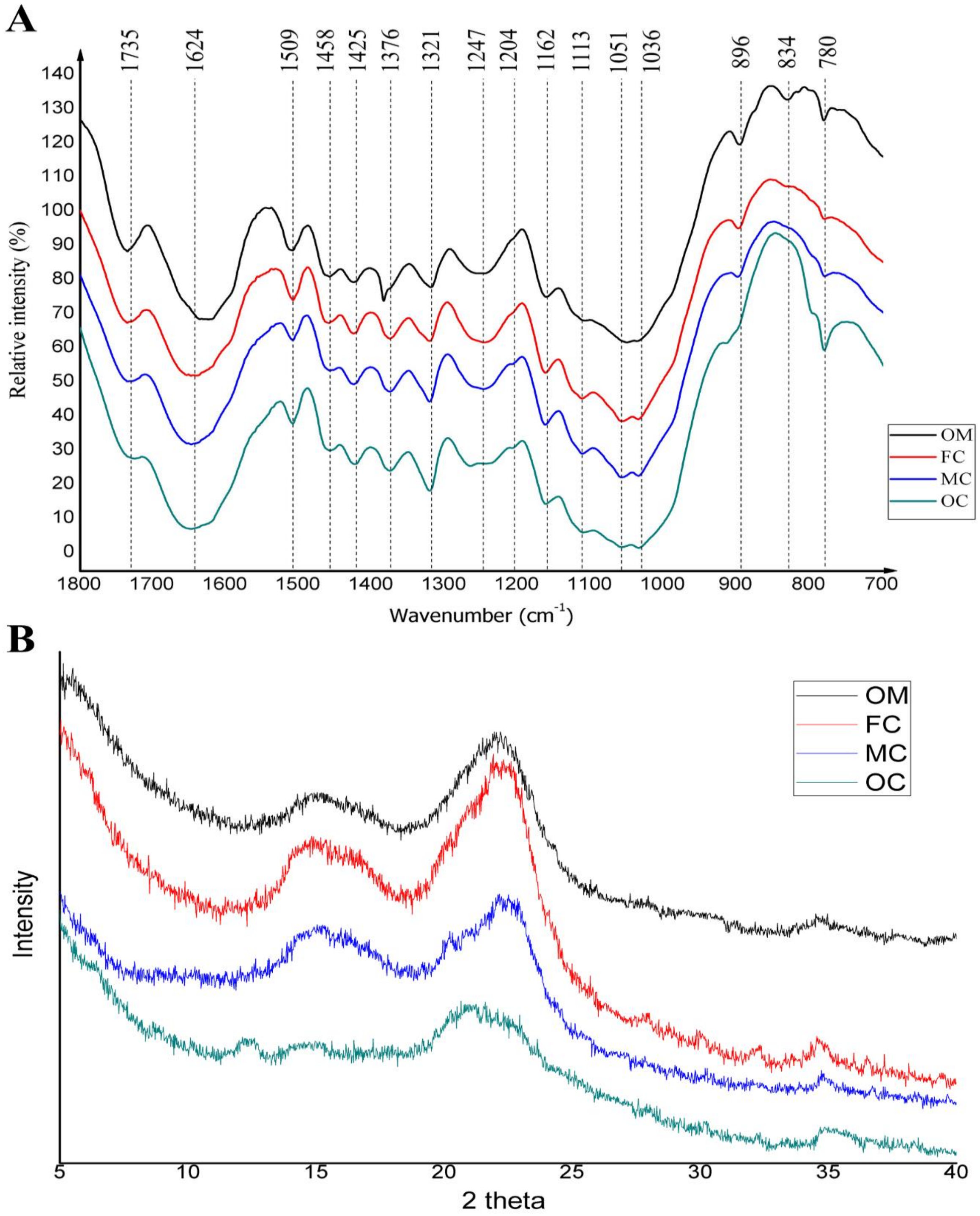

FIG S1. (A) FTIR spectra of original plant substrate and treated fungus combs. The common wavenumbers with corresponding bond vibrations and functional groups are listed in Table S2. (B) X-rays diffraction patterns of mulberry wood and all treated comb categories. OM: original mulberry wood; FC: fresh fungus comb; MC: mature fungus comb; OC: old fungus comb. N=3 colonies.

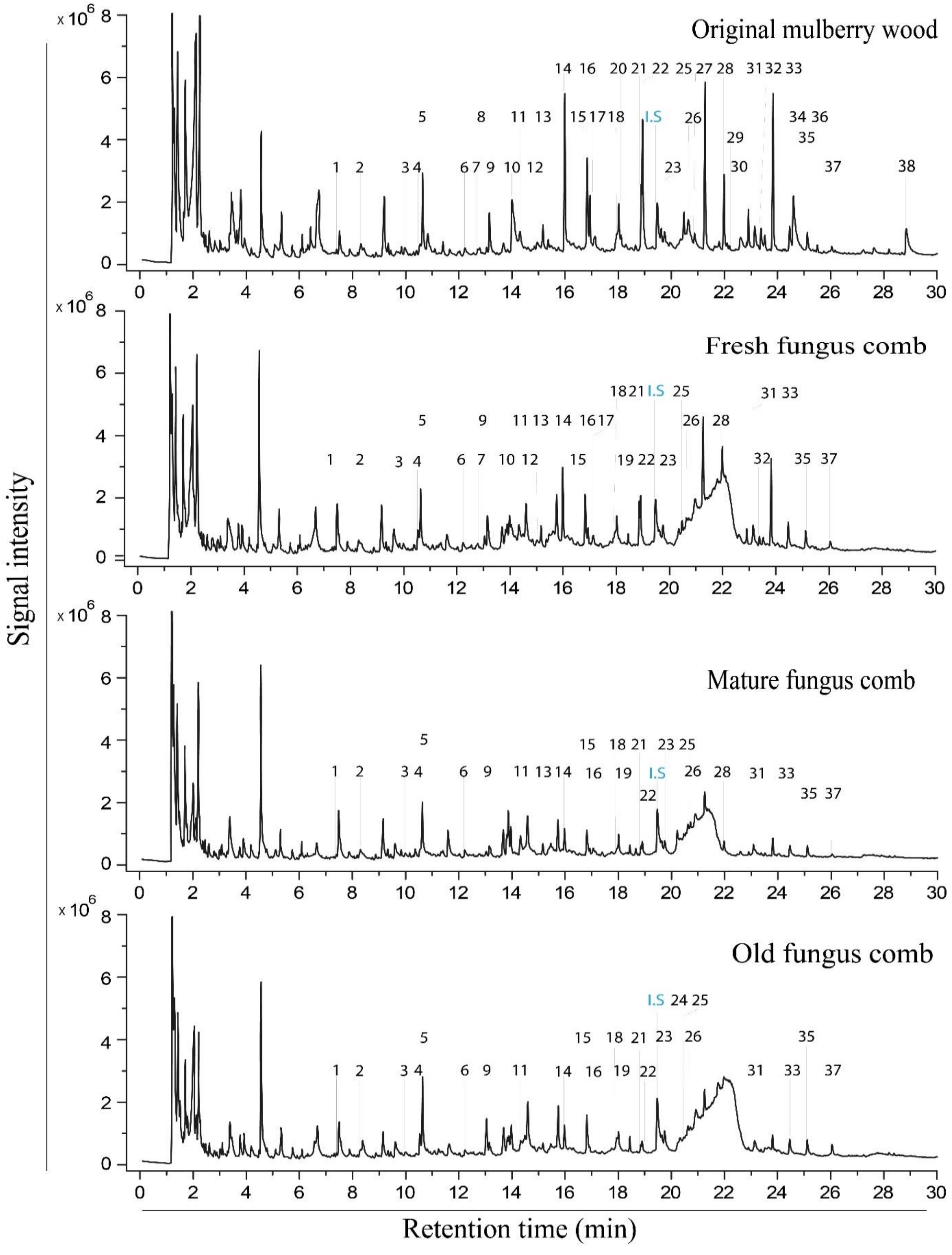

FIG S2. Py-GC/MS chromatograms of the original mulberry and treated three comb categories. The identities and relative abundances of lignin derived phenolic compounds released are listed in Fig. S3 and Table 1.

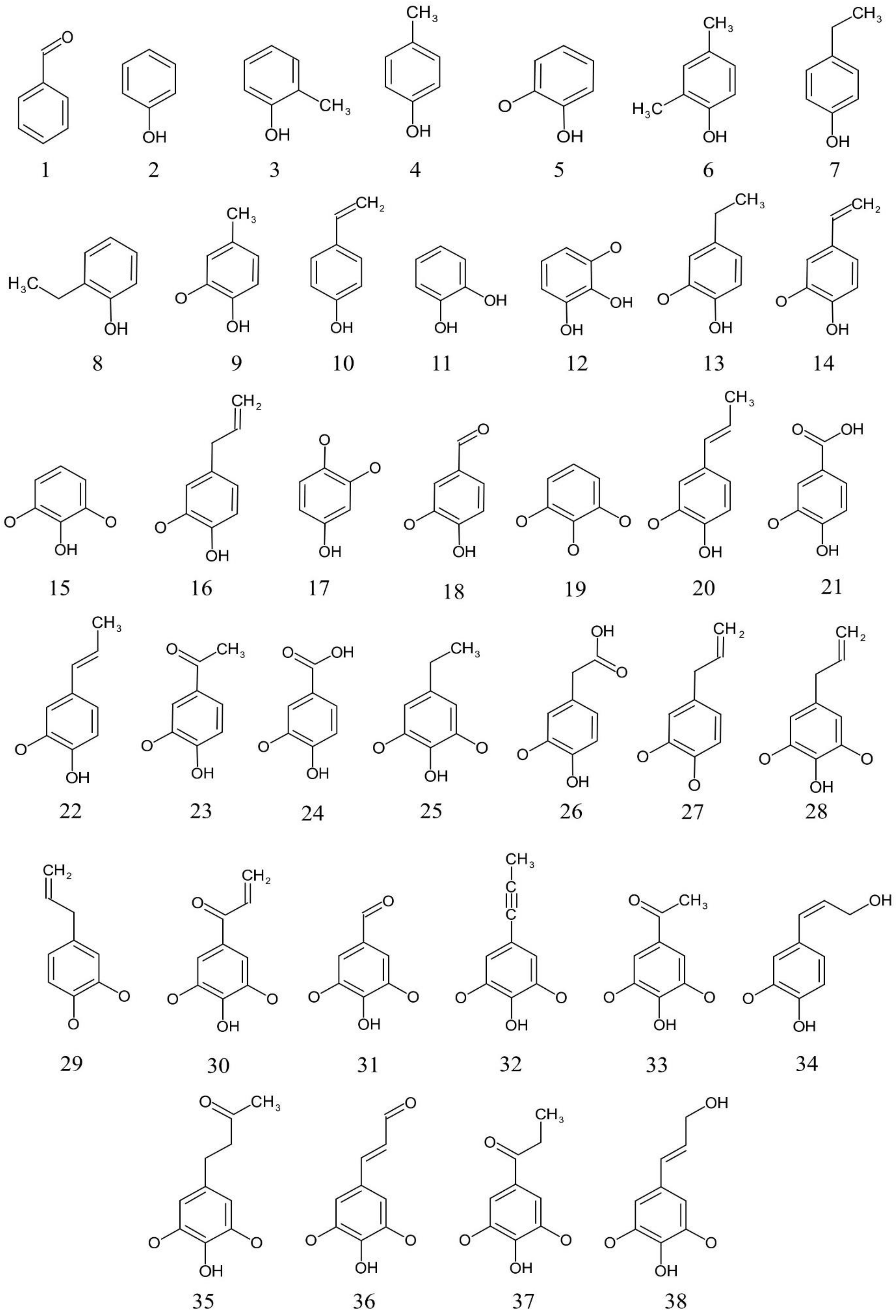

FIG S3. Structures of lignin derived phenolic compounds released from Py-GC/MS.

References

1. Sills DL, Gossett JM. 2012. Using FTIR to predict saccharification from enzymatic hydrolysis of alkali-pretreated biomasses. Biotechnol Bioeng 109:353-362. https://doi.org/10.1002/bit.23314.
2. Bekiaris G, Triolo JM, Peltre C, Pedersen L, Jensen LS, Bruun S. 2015. Rapid estimation of the biochemical methane potential of plant biomasses using Fourier transform mid-infrared photoacoustic spectroscopy. Bioresour Technol 197:475-481. https://doi.org/10.1016/j.biortech.2015.08.050.
3. Acquah GE, Via BK, Fasina OO, Eckhardt LG. 2016. Rapid quantitative analysis of forest biomass using fourier transform infrared spectroscopy and partial least squares regression. J Anal Methods Chem 2016. https://doi.org/10.1155/2016/1839598.
4. Stachowiak-Wencek A, Zborowska M, Waliszewska H, Waliszewska B. 2019. Chemical changes in lignocellulosic biomass (corncob) influenced by pretreatment and anaerobic digestion (AD). Bioresources 14:8082-8099.
5. Bekiaris G, Koutrotsios G, Tarantilis PA, Pappas CS, Zervakis GI. 2020. FTIR assessment of compositional changes in lignocellulosic wastes during cultivation of *Cyclocybe cylindracea* mushrooms and use of chemometric models to predict production performance. J Mater Cycles Waste Manag 1-9. https://doi.org/10.1007/s10163-020-00995-7.
6. Pandey KK, Pitman AJ. 2003. FTIR studies of the changes in wood chemistry following decay by brown-rot and white-rot fungi. Int Biodeterior Biodegradation 52:151-160. https://doi.org/10.1016/S0964-8305(03)00052-0.
7. Li H, Lu J, Mo J. 2012. Physiochemical lignocellulose modification in the formosan subterranean termite *Coptotermes Formosanus* Shiraki (Isoptera: Rhinotermitidae) and potential uses in the production of biofuels. Bioresources, 7(1), pp.0675-0685.

8. Droussi Z, D'orazio V, Provenzano MR, Hafidi M, Ouatmane A. 2009. Study of the biodegradation and transformation of olive-mill residues during composting using FTIR spectroscopy and differential scanning calorimetry. J Hazard Mater 164:1281-1285. https://doi.org/10.1016/j.jhazmat.2008.09.081.
9. Ciolacu D, Ciolacu F, Popa VI. 2011. Amorphous cellulose—structure and characterization. Cellul Chem Technol 45:13-21.
10. Lv P, Almeida G, Perré P. 2015. TGA-FTIR analysis of torrefaction of lignocellulosic components (cellulose, xylan, lignin) in isothermal conditions over a wide range of time durations. Bioresources 10:4239-4251.
11. Lupoi JS, Gjersing E, Davis MF. 2015. Evaluating lignocellulosic biomass, its derivatives, and downstream products with Raman spectroscopy. Front Bioeng Biotechnol 3:50. https://doi.org/10.3389/fbioe.2015.00050.
12. da Costa RR, Hu H, Pilgaard B, Vreeburg SM, Schückel J, Pedersen KS, Kračun SK, Busk PK, Harholt J, Sapountzis P, Lange L. 2018. Enzyme activities at different stages of plant biomass decomposition in three species of fungus-growing termites. Appl Environ Microbiol 84:e01815-17. https://doi.org/10.1128/AEM.01815-17.
13. Li H, Yelle DJ, Li C, Yang M, Ke J, Zhang R, Liu Y, Zhu N, Liang S, Mo X, Ralph J. 2017. Lignocellulose pretreatment in a fungus-cultivating termite. Proc Natl Acad Sci U S A 114:4709-4714. https://doi.org/10.1073/pnas.1618360114.
